# Impaired SERCA2a phosphorylation causes diabetic cardiomyopathy through impinging on cardiac contractility and precursor protein processing

**DOI:** 10.1093/lifemeta/loac013

**Published:** 2022-07-28

**Authors:** Chao Quan, Sangsang Zhu, Ruizhen Wang, Jiamou Chen, Qiaoli Chen, Min Li, Shu Su, Qian Du, Minjun Liu, Hong-Yu Wang, Shuai Chen

**Affiliations:** MOE Key Laboratory of Model Animal for Disease Study, Department of Cardiology, Nanjing Drum Tower Hospital, The Affiliated Hospital of Nanjing University Medical School, Model Animal Research Center, School of Medicine, Nanjing University, Nanjing, Jiangsu 210061, China; State Key Laboratory of Pharmaceutical Biotechnology, Department of Endocrinology, Nanjing Drum Tower Hospital, Model Animal Research Center, School of Medicine, Nanjing University, Nanjing, Jiangsu 210061, China; MOE Key Laboratory of Model Animal for Disease Study, Department of Cardiology, Nanjing Drum Tower Hospital, The Affiliated Hospital of Nanjing University Medical School, Model Animal Research Center, School of Medicine, Nanjing University, Nanjing, Jiangsu 210061, China; State Key Laboratory of Pharmaceutical Biotechnology, Department of Endocrinology, Nanjing Drum Tower Hospital, Model Animal Research Center, School of Medicine, Nanjing University, Nanjing, Jiangsu 210061, China; MOE Key Laboratory of Model Animal for Disease Study, Department of Cardiology, Nanjing Drum Tower Hospital, The Affiliated Hospital of Nanjing University Medical School, Model Animal Research Center, School of Medicine, Nanjing University, Nanjing, Jiangsu 210061, China; State Key Laboratory of Pharmaceutical Biotechnology, Department of Endocrinology, Nanjing Drum Tower Hospital, Model Animal Research Center, School of Medicine, Nanjing University, Nanjing, Jiangsu 210061, China; MOE Key Laboratory of Model Animal for Disease Study, Department of Cardiology, Nanjing Drum Tower Hospital, The Affiliated Hospital of Nanjing University Medical School, Model Animal Research Center, School of Medicine, Nanjing University, Nanjing, Jiangsu 210061, China; State Key Laboratory of Pharmaceutical Biotechnology, Department of Endocrinology, Nanjing Drum Tower Hospital, Model Animal Research Center, School of Medicine, Nanjing University, Nanjing, Jiangsu 210061, China; MOE Key Laboratory of Model Animal for Disease Study, Department of Cardiology, Nanjing Drum Tower Hospital, The Affiliated Hospital of Nanjing University Medical School, Model Animal Research Center, School of Medicine, Nanjing University, Nanjing, Jiangsu 210061, China; State Key Laboratory of Pharmaceutical Biotechnology, Department of Endocrinology, Nanjing Drum Tower Hospital, Model Animal Research Center, School of Medicine, Nanjing University, Nanjing, Jiangsu 210061, China; MOE Key Laboratory of Model Animal for Disease Study, Department of Cardiology, Nanjing Drum Tower Hospital, The Affiliated Hospital of Nanjing University Medical School, Model Animal Research Center, School of Medicine, Nanjing University, Nanjing, Jiangsu 210061, China; State Key Laboratory of Pharmaceutical Biotechnology, Department of Endocrinology, Nanjing Drum Tower Hospital, Model Animal Research Center, School of Medicine, Nanjing University, Nanjing, Jiangsu 210061, China; MOE Key Laboratory of Model Animal for Disease Study, Department of Cardiology, Nanjing Drum Tower Hospital, The Affiliated Hospital of Nanjing University Medical School, Model Animal Research Center, School of Medicine, Nanjing University, Nanjing, Jiangsu 210061, China; State Key Laboratory of Pharmaceutical Biotechnology, Department of Endocrinology, Nanjing Drum Tower Hospital, Model Animal Research Center, School of Medicine, Nanjing University, Nanjing, Jiangsu 210061, China; MOE Key Laboratory of Model Animal for Disease Study, Department of Cardiology, Nanjing Drum Tower Hospital, The Affiliated Hospital of Nanjing University Medical School, Model Animal Research Center, School of Medicine, Nanjing University, Nanjing, Jiangsu 210061, China; State Key Laboratory of Pharmaceutical Biotechnology, Department of Endocrinology, Nanjing Drum Tower Hospital, Model Animal Research Center, School of Medicine, Nanjing University, Nanjing, Jiangsu 210061, China; MOE Key Laboratory of Model Animal for Disease Study, Department of Cardiology, Nanjing Drum Tower Hospital, The Affiliated Hospital of Nanjing University Medical School, Model Animal Research Center, School of Medicine, Nanjing University, Nanjing, Jiangsu 210061, China; State Key Laboratory of Pharmaceutical Biotechnology, Department of Endocrinology, Nanjing Drum Tower Hospital, Model Animal Research Center, School of Medicine, Nanjing University, Nanjing, Jiangsu 210061, China; MOE Key Laboratory of Model Animal for Disease Study, Department of Cardiology, Nanjing Drum Tower Hospital, The Affiliated Hospital of Nanjing University Medical School, Model Animal Research Center, School of Medicine, Nanjing University, Nanjing, Jiangsu 210061, China; State Key Laboratory of Pharmaceutical Biotechnology, Department of Endocrinology, Nanjing Drum Tower Hospital, Model Animal Research Center, School of Medicine, Nanjing University, Nanjing, Jiangsu 210061, China; Jiangsu Key Laboratory of Molecular Medicine, Model Animal Research Center, School of Medicine, Nanjing University, Nanjing 210061, China; MOE Key Laboratory of Model Animal for Disease Study, Department of Cardiology, Nanjing Drum Tower Hospital, The Affiliated Hospital of Nanjing University Medical School, Model Animal Research Center, School of Medicine, Nanjing University, Nanjing, Jiangsu 210061, China; State Key Laboratory of Pharmaceutical Biotechnology, Department of Endocrinology, Nanjing Drum Tower Hospital, Model Animal Research Center, School of Medicine, Nanjing University, Nanjing, Jiangsu 210061, China; Jiangsu Key Laboratory of Molecular Medicine, Model Animal Research Center, School of Medicine, Nanjing University, Nanjing 210061, China

**Keywords:** SERCA, SPEG, phosphorylation, calcium, insulin, diabetic cardiomyopathy

## Abstract

Diabetic cardiomyopathy (DCM) is currently a progressive and nonstoppable complication in type 2 diabetic patients. Metabolic insults and insulin resistance are involved in its pathogenesis; however, the underlying mechanisms are still not clearly understood. Here we show that calcium dysregulation can be both a cause and a consequence of cardiac insulin resistance that leads to DCM. A western diet induces the development of DCM through at least three phases in mice, among which an early phase depends on impaired Thr^484^-phosphorylation of sarcoplasmic/endoplasmic reticulum calcium ATPase 2a (SERCA2a) elicited by insulin resistance. Mutation of SERCA2a-Thr^484^ to a nonphosphorylatable alanine delays calcium re-uptake into the sarcoplasmic reticulum in the cardiomyocytes and decreases cardiac function at the baseline. Importantly, this mutation blunts the early phase of DCM, but has no effect on disease progression in the following phases. Interestingly, impairment of sarcoplasmic reticulum calcium re-uptake caused by the SERCA2a-Thr^484^ mutation inhibited processing of insulin receptor precursor through FURIN convertase, resulting in cardiac insulin resistance. Collectively, these data reveal a bidirectional relationship between insulin resistance and impairment of calcium homeostasis, which may underlie the early pathogenesis of DCM. Our findings have therapeutic implications for early intervention of DCM.

## Introduction

Diabetic cardiomyopathy (DCM) is a progressive complication associated with type 2 diabetes (T2D), causing deterioration of cardiac function in diabetic patients, independent of coronary artery disease, and hypertension [1]. World-wide prevalence of T2D urges a better understanding of molecular mechanisms underlying the pathogenesis of DCM.

Contractile properties of cardiomyocytes, whose pathological alterations contribute to the development of DCM, are influenced by both metabolic and hormonal factors [2]. For instance, hyperglycemia and dyslipidemia in T2D result in cardiac toxicity that induces oxidative stress and impairs mitochondria function [3]. The heart is an insulin-sensitive organ in which myocardial insulin resistance impairs glucose metabolism and causes fuel shift toward fatty acid oxidation in cardiomyocytes [4]. This substrate shift dampens metabolic flexibility in diabetic heart [4]. Besides these metabolic changes, myocardial insulin resistance also causes dysregulation of Ca^2+^ homeostasis in cardiomyocytes [5]. In turn, elevations in cytosolic Ca^2+^ might aggregate insulin resistance through yet unclear mechanisms [6]. It is currently unclear how these metabolic assaults, impaired insulin action and abnormal cellular Ca^2+^ homeostasis temporally contribute to the pathogenesis of DCM.

Ca^2+^ homeostasis in cardiomyocytes involves its cycling between the cytosol and sarcoplasmic reticulum (SR) during excitation–contraction coupling, which consequently controls contraction and relaxation of cardiac muscle [7]. Re-uptake of Ca^2+^ from the cytosol into the SR is mediated by sarcoplasmic/endoplasmic reticulum Ca^2+^ ATPase 2a (SERCA2a), whose dysfunction is a hallmark of heart failure [8, 9]. Upon stimulation with insulin, SERCA2a in the heart is phosphorylated on its Thr^484^ by the striated muscle preferentially expressed protein kinase (SPEG) that itself is phosphorylated by protein kinase B (PKB, also known as Akt) [10, 11]. SPEG contains two Ser/Thr kinase domains, namely SK1 and SK2 [12], and PKB-mediated phosphorylation activates the SK2 domain of SPEG, which consequently phosphorylates SERCA2a-Thr^484^ [11]. Myocardial insulin resistance elicited by the cardiac-specific PKB inactivation impairs phosphorylation of SPEG and SERCA2a, and results in cardiac dysfunction [11]. Moreover, a knockin mutation preventing PKB-mediated SPEG phosphorylation inactivates its SK2 domain, decreases SERCA2a-Thr^484^ phosphorylation, and impairs cardiac function [11]. However, the PKB−SPEG pathway may regulate cardiac function through phosphorylation of protein substrates other than SERCA2a. Therefore, the precise role of SERCA2a-Thr^484^ phosphorylation *in vivo* is still to be defined, particularly regarding its roles in the pathogenesis of myocardial insulin resistance and DCM.

In this study, we investigated the pathophysiological mechanisms of DCM involving SERCA2a-Thr^484^ phosphorylation using a knockin mouse model. We utilized this mouse model and their derived cardiomyocytes to reveal potential roles of SERCA2a in the pathogenesis of myocardial insulin resistance and DCM.

## Results

### A western diet decreases insulin-induced SERCA2a-Thr^484^ phosphorylation and impairs cardiac function

**Figure 1:**
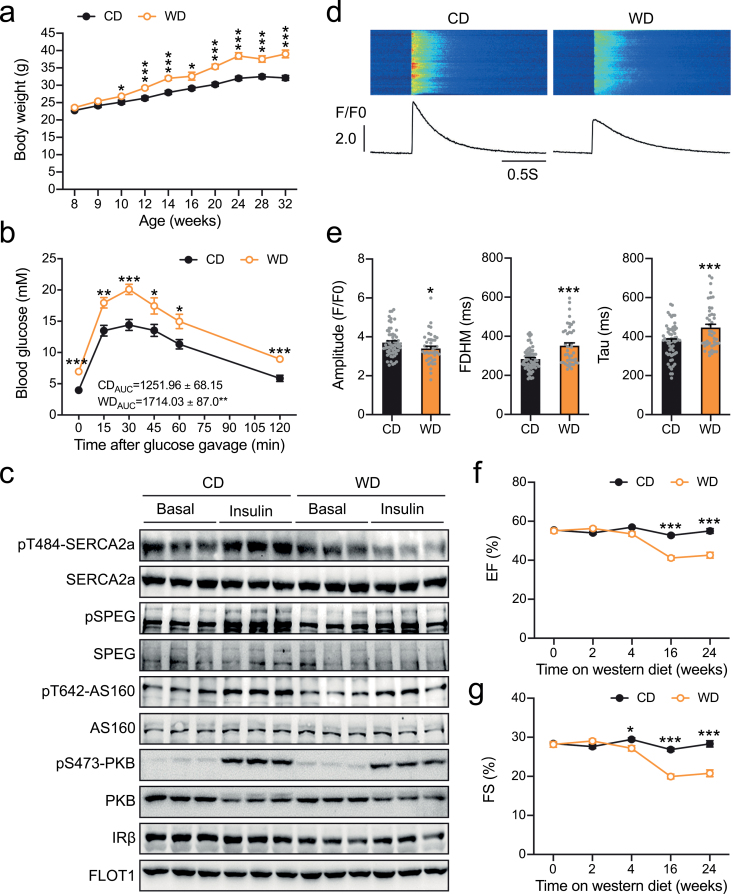
Effects of a WD on cardiac function and Ca^2+^ homeostasis in primary cardiomyocytes. (a) Body weights of male C57/Bl6J mice fed with a CD or a WD. *n* = 9−12. (b) Glucose tolerance test of male C57/Bl6J mice at 24 weeks of age. The mice were fed with WD for 16 weeks. The values show the glucose area under the curve during the glucose tolerance test. AUC, area under the curve. *n* = 7−8. (c) Expression and phosphorylation of SERCA2a, SPEG, AS160, PKB, and IRβ in primary cardiomyocytes isolated from CD- or WD-fed male mice (10-month-old, fasted overnight). The mice were fed with WD for 32 weeks. Primary cardiomyocytes were stimulated with or without insulin (300 nM, 30 min) before lysed for immunoblotting analyses. (d and e) Ca^2+^ transients elicited by electrical stimulation (0.5 Hz) in primary cardiomyocytes isolated from the CD- or WD-fed male mice (7-month-old, fed ad libitum). The mice were fed with WD for 20 weeks. (d) Representative Ca^2+^ transient images and curves. (e) Amplitudes, FDHM and time constant Tau of Ca^2+^ transients. Sixty cells from 2 CD-fed mice and 43 cells from 2 WD-fed mice were analyzed. (f and g) EF (f) and FS (g) measured via echocardiography in CD- or WD-fed C57/Bl6J male mice (WD feeding started at the age of 2-month). *n* = 6–12. The data are given as the mean ± SEM. Statistical analyses were carried out using Student’s *t*-test. * indicates *P* < 0.05, **P < 0.01, and ***P < 0.001.

Western diet (WD) causes systemic insulin resistance and results in DCM [13]. As expected, mice on a WD became obese, and developed systemic insulin resistance as evidenced by impaired glucose tolerance (Fig. 1a and b). Insulin receptor β subunit (IRβ) was decreased in primary cardiomyocytes isolated from WD-fed mice as compared to that in cells from mice fed with a normal chow diet (CD) (Fig. 1c, Supplementary Fig. S1a). Accordingly, insulin-stimulated phosphorylation of PKB and its substrate AS160 was decreased in primary cardiomyocytes from WD-fed mice (Fig. 1c, Supplementary Fig. S1d and e). Insulin regulates phosphorylation of SERCA2a-Thr^484^ through the PKB-SPEG pathway [11]. In agreement with the impaired PKB activation, insulin-stimulated phosphorylation of SPEG and SERCA2a-Thr^484^ was blunted in primary cardiomyocytes from WD-fed mice (Fig. 1c, Supplementary Fig. S1b and c). We then measured Ca^2+^ transient in primary cardiomyocytes, and found that it was depressed in cells from WD-fed mice (Fig. 1d and e). The full duration at half maximum (FDHM) and time constant Tau were both increased in cardiomyocytes from WD-fed mice (Fig. 1d and e), suggesting an impairment of Ca^2+^ re-uptake into SR. Cardiac function of WD-fed mice exhibited a progressive decline with enlarged volumes of left ventricles (LV) and thin ventricular walls (Fig. 1f and g, Supplementary Fig. S1f). Together, these data show that WD-induced DCM is accompanied with an impairment of SERCA2a-Thr^484^ phosphorylation due to cardiac insulin resistance.

### Generation and basic characterization of a SERCA2a^Thr484Ala^ knockin mouse model

**Figure 2:**
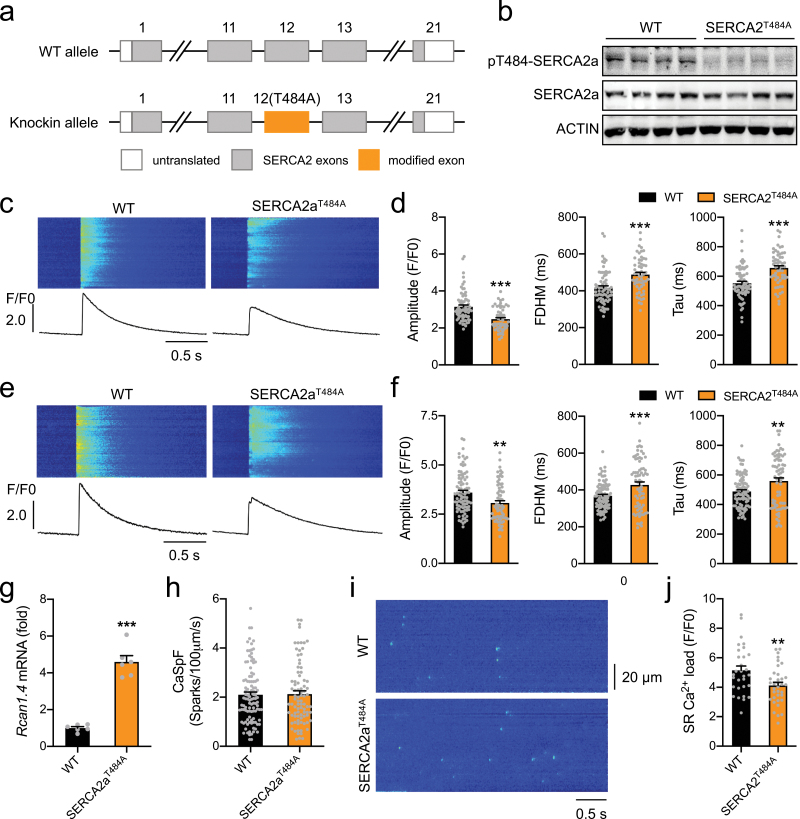
Ca^2+^ homeostasis in cardiomyocytes of the SERCA2a^T484A^ mice. (a) Diagrammatic illustration of the WT and T484A knockin allele of SERCA2a. The Thr^484^ residue on SERCA2a was changed to alanine via CRISPR/Cas9-assisted knockin substitution to generate SERCA2a^T484A^ knockin mice. (b) SERCA2a-Thr^484^ phosphorylation in cardiomyocytes of the WT and SERCA2a^T484A^ female mice (2-month-old, fed ad libitum). (c and d) Ca^2+^ transients elicited by electrical stimulation in primary cardiomyocytes isolated from the WT and SERCA2a^T484A^ mice (3-month-old, male and female, fed ad libitum). (c) Representative Ca^2+^ transient images and curves. (d) Quantification of amplitudes, FDHM and Tau of Ca^2+^ transients. Seventy cells from 3 WT mice and 57 cells from 3 SERCA2a^T484A^ mice were analyzed. (e and f) Ca^2+^ transients elicited by electrical stimulation in primary cardiomyocytes isolated from the WT and SERCA2a^T484A^ mice (13-month-old, male, fed ad libitum). (e) Representative Ca^2+^ transient images and curves. (f) Quantification of amplitudes, FDHM and Tau of Ca^2+^ transients. Eighty-eight cells from 2 WT mice and 71 cells from 2 SERCA2a^T484A^ mice were analyzed. (g) Expression of *Rcan1.4* mRNA in primary cardiomyocytes from the WT and SERCA2a^T484A^ mice (2-month-old, male). *n* = 6. (h and i) Ca^2+^ sparks in primary cardiomyocytes isolated from the WT and SERCA2a^T484A^ mice (17-month-old, male, fed ad libitum). (h) Quantification of Ca^2+^ spark frequency. Eighteen cells from 1 WT mice and 15 cells from 1 knockin mice were analyzed. (i) Representative images of Ca^2+^ sparks. (j) SR Ca^2+^ load in primary cardiomyocytes isolated from the WT and SERCA2a^T484A^ mice (4-month-old, female, fed ad libitum). Thirty cells from 5 WT mice and 35 cells from 6 knockin mice were analyzed. The data are given as the mean ± SEM. Statistical analyses were carried out using Student’s *t*-test. ** indicates *P* < 0.01 and ***P < 0.001.

To elucidate the precise role of SERCA2a-Thr^484^ phosphorylation in the development of DCM, we gengerated a mouse model in which SERCA2a-Thr^484^ was mutated to a nonphosphorylatable alanine through a Cas9-mediated site mutagenesis (Fig. 2a, Supplementary Fig. S2a and b). The knockin mutation did not affect expression of SERCA2a and its upstream kinase SPEG in the heart (Supplementary Fig. S2c and d). As expected, this mutation prevented phosphorylation of SERCA2a recognized by the phospho-Thr^484^ antibody in cardiomyocytes of the knockin mice (Fig. 2b). The SERCA2a^Thr484Ala^ knockin mice were viable and displayed no overt phenotype. These mice had normal fasting blood glucose levels and intact ability to clear blood glucose after administration with a bolus of glucose as compared to their wild-type (WT) littermates (Supplementary Fig. S2e−h). Cardiac expression of genes encoding key enzymes in glucose and fatty acid metabolism was comparable between the SERCA2a^Thr484Ala^ mice and WT littermates, including *Glut1*, *Glut4*, *Hk2*, *Pfk2*, *Lpl*, *Cd36*, *Fatp1*, *Fabp3*, *Atgl,* and *Cpt1* (Supplementary Fig. S2i). Similarly, protein levels of GLUT1, GLUT4, and CD36 remained normal in the heart of SERCA2a^Thr484Ala^ mice (Supplementary Fig. S2c and d). Together, these data demonstrate that the SERCA2a^Thr484Ala^ knockin mice and their derived cells are useful reagents for investigation of the function of SERCA2a-Thr^484^ phosphorylation.

### The SERCA2a^Thr484Ala^ knockin mutation impairs Ca^2+^ homeostasis in cardiomyocytes

SERCA2a is a critical regulator of Ca^2+^ homeostasis, whose Thr^484^ phosphorylation has been implicated in regulation of Ca^2+^ re-uptake into SR in cardiomyocytes [10]. We isolated primary cardiomyocytes from young (3-month-old) and old (13-month-old) mice and measured Ca^2+^ transients using a Fluo-4 based assay upon a low frequency (0.5 Hz) of electrical stimulation. The FDHM and Tau were significantly increased in cardiomyocytes from young SERCA2a^Thr484Ala^ mice as compared to those in WT cells (Fig. 2c and d), suggesting that the knockin mutation prolonged Ca^2+^ re-uptake into SR. The delay of SR Ca^2+^ re-uptake might result in elevation of cytosolic Ca^2+^. Indeed, the amplitude of Ca^2+^ transients, a measure of cytosolic Ca^2+^, was significantly decreased in SERCA2a^Thr484Ala^ cardiomyocytes as compared to that in WT cells (Fig. 2c and d), suggesting that cytosolic Ca^2+^ was elevated in SERCA2a^Thr484Ala^ cardiomyocytes. Such effects of the SERCA2a^Thr484Ala^ knockin mutation on Ca^2+^ homeostasis persisted in cardiomyocytes from old mice (Fig. 2e and f). We also measured Ca^2+^ transients in primary cardiomyocytes from the SERCA2a^Thr484Ala^ mice upon a high frequency (3 Hz) of electrical stimulation. Ca^2+^ transients were also depressed with a prolonged period of Ca^2+^ re-uptake into SR in SERCA2a^Thr484Ala^ cardiomyocytes upon the high frequency (3 Hz) of electrical stimulation (Supplementary Fig. S3a−c), which were similar to the phenotypic changes upon the low frequency (0.5 Hz) of electrical stimulation (Supplementary Fig. S3d−f). Cytosolic Ca^2+^ is a critical regulator of gene expression. In agreement with elevation of cytosolic Ca^2+^, mRNA level of *Rcan1.4*, a Ca^2+^-responsive gene, was significantly increased in cardiomyocytes of the SERCA2a^Thr484Ala^ knockin mice (Fig. 2g). Phospholamban is a key regulator of SERCA2a, whose expression and phosphorylation were comparable between the WT and SERCA2a^Thr484Ala^ knockin mice (Supplementary Fig. S3g−j).

We next examined Ca^2+^ sparks in primary cardiomyocytes, and found that their frequency was comparable in the two genotypes (Fig. 2h and i), suggesting that RyR2-mediated spontaneous Ca^2+^ release from SR was most likely unaltered. In agreement, expression and phosphorylation of RyR2 were normal in the heart of SERCA2a^Thr484Ala^ knockin mice (Supplementary Fig. S3g and h, k−m). Probably as a result of defective SR Ca^2+^ re-uptake, SR Ca^2+^ load was significantly decreased in SERCA2a^Thr484Ala^ cardiomyocytes as compared to WT control cells (Fig. 2j).

Taken together, these data demonstrate that the SERCA2a^Thr484Ala^ knockin mutation impairs Ca^2+^ homeostasis in cardiomyocytes.

### Abnormal cellular Ca^2+^ handling causes insulin resistance in SERCA2a^Thr484Ala^ cardiomyocytes

**Figure 3:**
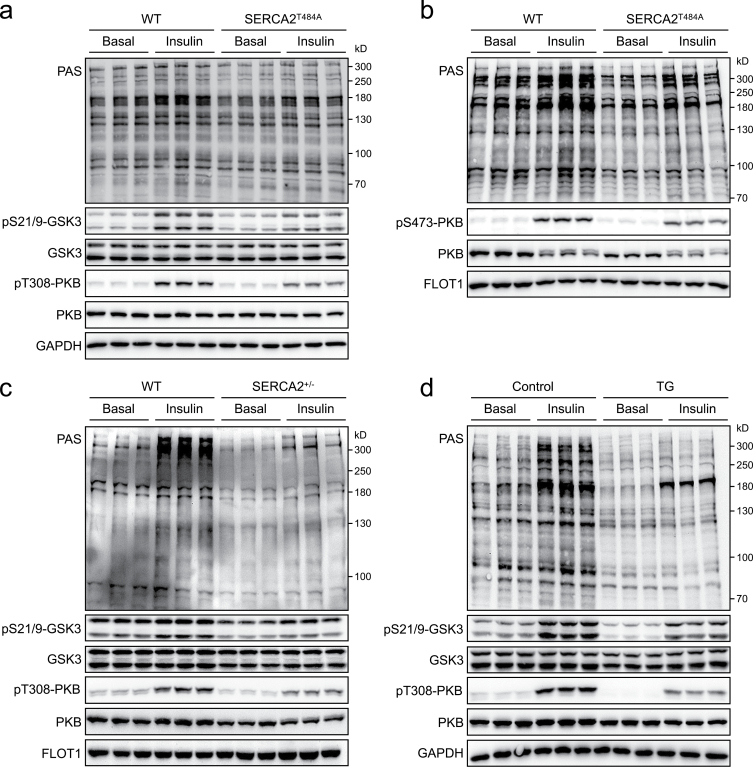
Insulin signaling in primary cardiomyocytes upon SERCA2a inhibition. (a) Insulin signaling in primary cardiomyocytes isolated from the neonatal WT or SERCA2a^T484A^ knockin mice (male and female). Cardiomyocytes were stimulated with or without insulin (300 nM, 30 min). Phosphorylation of PKB and GSK3, and PAS-reactive phosphorylation were detected via immunoblotting using GAPDH as a loading control. (b) Insulin signaling in primary cardiomyocytes isolated from the adult WT or SERCA2a^T484A^ knockin mice (15-month-old, male, fasted overnight). Cardiomyocytes were stimulated with or without insulin (300 nM, 30 min). Phosphorylation of PKB and PAS-reactive phosphorylation were detected via immunoblotting using FLOT1 as a loading control. (c) Insulin signaling in primary cardiomyocytes isolated from the adult WT or SERCA2a^+/−^ mice (3-month-old, male, fasted overnight). Cardiomyocytes were stimulated with or without insulin (300 nM, 30 min). Phosphorylation of PKB and GSK3, and PAS-reactive phosphorylation were detected via immunoblotting using FLOT1 as a loading control. (d) Insulin signaling in control or TG-pretreated NRVCs. NRVCs were pretreated with or without 2 μM TG for 24 h, and then stimulated with or without insulin (300 nM) for 30 min. Phosphorylation of PKB and GSK3, and PAS-reactive phosphorylation were detected via immunoblotting using GAPDH as a loading control.

We next examined whether, and if so how, the SERCA2a^Thr484Ala^ knockin mutation impacts on insulin signaling in cardiomyocytes. To this end, primary cardiomyocytes were isolated from the neonatal and adult WT and SERCA2a^Thr484Ala^ knockin mice. Interestingly, insulin-stimulated phosphorylation of PKB was attenuated in neonatal SERCA2a^Thr484Ala^ knockin cardiomyocytes as compared to WT cells (Fig. 3a). In agreement with the lower PKB activation, insulin-induced phosphorylation of PKB substrates detected by a phospho-(ser/thr) AKT substrate (PAS) antibody was substantially decreased in neonatal SERCA2a^Thr484Ala^ knockin cardiomyocytes (Fig. 3a). GSK3 is a PKB substrate, whose phosphorylation in response to insulin stimulation was also lower in neonatal SERCA2a^Thr484Ala^ knockin cardiomyocytes than in WT cells (Fig. 3a). Similar inhibition of the insulin-PKB signaling was observed in cardiomyocytes of the adult SERCA2a^Thr484Ala^ knockin mice (Fig. 3b). The development of insulin resistance in the SERCA2a^Thr484Ala^ knockin cardiomyocytes suggests that SERCA2a is not only a target of the insulin−PKB pathway but also a critical regulator of myocardial insulin signaling.

We then investigated whether abnormal Ca^2+^ handling causes insulin resistance in cardiomyocytes. It has been reported that heterozygous deletion of SERCA2a impairs cardiac performance [14]. We therefore took another genetic approach to inhibit SERCA2a function in the heart by deletion of one allele of *Serca2a* in mice through a Cas9-mediated mutagenesis (Supplementary Fig. S4a and b). SERCA2a was decreased by 50% at the mRNA level and 30% at the protein level in the heart of the heterozygous SERCA2a knockout mice (Supplementary Fig. S4c–e). Similarly as the SERCA2a^Thr484Ala^ knockin mutation, heterozygosity deficiency of SERCA2a weakened insulin-stimulated phosphorylation of PKB, decreased the PAS-reactive phosphorylation of PKB substrates, and lowered phosphorylation of GSK3 in primary cardiomyocytes (Fig. 3c). We then took a pharmacological approach to inhibit SERCA2a by thapsigargin (TG) in neonatal rat ventricular cardiomyocytes (NRVCs). Again, pharmacological inhibition of SERCA2a strongly blocked PKB phosphorylation in response to insulin, which was accompanied with marked decreases of insulin-stimulated GSK3 phosphorylation and PAS-reactive phosphorylation of PKB substrates in NRVCs (Fig. 3d). Unlike TG that inhibits SR Ca^2+^ re-uptake, Ca^2+^ inophore A23187 mobilizes Ca^2+^ from SR into the cytosol [15]. Despite this difference in their action modes, the two compounds exert similar effects on cellular Ca^2+^ homeostasis. Importantly, A23187 caused insulin resistance similar to TG when added to NRVCs or H9C2 cardiomyocytes (Supplementary Fig. S5a and b).

Together, these data show that abnormal Ca^2+^ handling causes insulin resistance in SERCA2a^Thr484Ala^ knockin cardiomyocytes.

### The SERCA2a^Thr484Ala^ mutation impairs precursor protein processing through FURIN in cardiomyocytes

**Figure 4:**
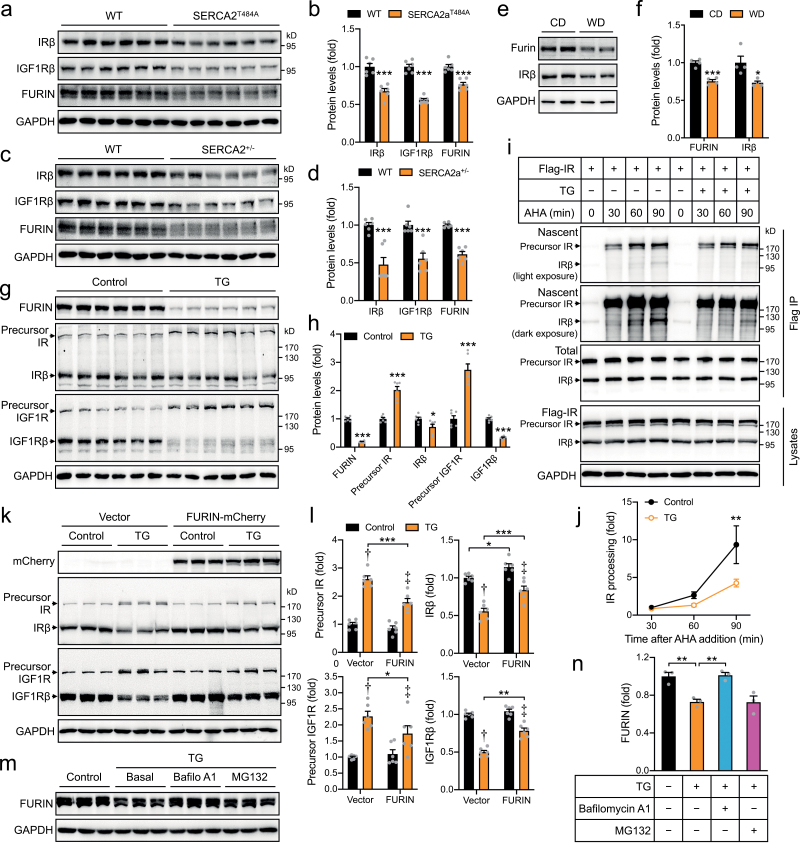
FURIN-dependent protein precursor processing in cells with impaired Ca^2+^ homeostasis. (a, b) Expression of FURIN, IRβ, and IGF1Rβ in primary cardiomyocytes of the WT and SERCA2a^T484A^ mice (2-month-old, male). GAPDH was used as a loading control. (a) Immunoblots. (b) Quantification of immunoblot signals. *n* = 6. (c and d) Expression of FURIN, IRβ, and IGF1Rβ in primary cardiomyocytes of the WT and SERCA2a^+/−^ mice (3-month-old, male). GAPDH was used as a loading control. (c) Immunoblots. (d) Quantification of immunoblot signals. *n* = 6. (e and f) Expression of FURIN and IRβ in the heart of CD- or WD-fed mice (12-month-old, male). The mice were fed with WD for 40 weeks. (e) Representative immunoblots. (f) Quantification of immunoblot signals. *n* = 4. (g, h) Expression of FURIN, precursor IR, IRβ, precursor IGF1R, and IGF1Rβ in NRVCs stimulated with or without TG (2 μM, 24h). (g) Immunoblots. (h) Quantification of immunoblot signals. *n* = 6. (I, j) IR processing in cells upon treatment with TG. HEK293 cells transfected with Flag-IR were treated with Click-iT^TM^ AHA for the indicated time in the presence or absence of TG (2 μM, 90 min). After immunoprecipitation using the Flag beads, nascent precursor IR, and mature IRβ were reacted with Click-iT^TM^ Protein Reaction Buffer Kit, and detected using HRP-labeled Avidin antibody. (i) Representative blots. (j) Quantification of IR processing that was defined as mature IRβ relative to precursor IR. *n* = 5. (k, l) Effects of FURIN expression on IR and IGF1R processing in TG-treated HEK293 cells. FURIN-mCherry or free mCherry was expressed in HEK293 cells that were stimulated with or without TG (2 μM) for 24 h. FURIN, precursor IR, IRβ, precursor IGF1R, and IGF1Rβ proteins were detected in cell lysates via immunoblotting. (k) Representative blots. (l) Quantitative data. *n* = 6. † indicates *P* < 0.001 (TG vector vs Control vector), and ‡ indicates *P* < 0.001 (TG FURIN-mCherry vs Control FURIN-mCherry) except for precursor IGF1R where ‡ indicates *P* < 0.05 (TG FURIN-mCherry vs Control FURIN-mCherry). (m, n) FURIN protein levels in HEK293 cells that were treated with the proteosome inhibitor MG-132 (10 μM) or the lysosome inhibitor Bafilomycin A1 (400 nM) in the presence of TG (2 μM) for 4 h. (m) Representative blots. (n) Quantitative data. *n* = 3. The data are given as the mean ± SEM. Statistical analyses were carried out via Student’s *t*-test for (b), (d), (f), and (h), via one-way ANOVA for (n), and via two-way ANOVA for (l) and (j). * indicates *P* < 0.05, **P < 0.01, and ***P < 0.001.

Insulin resistance in cardiomyocytes with impaired Ca^2+^ homeostasis made us examine expression of insulin receptor (IR) that consists of α and β subunits processed from a common precursor. Interestingly, IRβ was decreased in the SERCA2a^Thr484Ala^ and SERCA2a^+/−^ cardiomyocytes as compared to those in WT cells (Fig. 4a−d). The decrease of IRβ in the SERCA2a^Thr484Ala^ and SERCA2a^+/−^ cardiomyocytes was the most likely caused by lower SR Ca^2+^ due to inhibition of SR Ca^2+^ re-uptake. In agreement with this notion, treatments with TG or A23187 also caused a diminution of IRβ but an increase of IR precursor in cardiomyocytes (Fig. 4g and h, Supplementary Fig. S5c and d). These data show that abnormal Ca^2+^ handling in SR might impair processing of IR precursor in cardiomyocytes. Indeed, a pulse-chase experiment monitoring IR processing showed that TG treatment markedly inhibited production of IRβ from its precursor (Fig. 4i and j). Proprotein convertase FURIN is a key enzyme in cleaving IR precursor to produce IRα and IRβ [16], whose maturation as well as enzymatic activity depends on Ca^2+^ [17]. Interestingly, FURIN protein was markedly decreased in the SERCA2a^Thr484Ala^ as well as SERCA2a^+/−^ cardiomyocytes (Fig. 4a−d). Moreover, FURIN protein was also significantly lowered in the heart of WD-fed mice (Fig. 4e and f), in concomitance with decreases of IRβ (Fig. 4e and f). FURIN protein was also diminished in HEK293 cells treated with TG or A23187 in concomitance with impaired IR processing (Fig. 4g and h, Supplementary Fig. S5c and d). TG- or A23187-induced decrease of FURIN protein occurred as early as 30 min after additions into cells, ahead of inhibition of IR processing (Supplementary Figs. 5e and f, 6a and b). The diminution of FURIN was the most likely responsible for the impairment of IR processing since overexpression of FURIN reversed the inhibition of IR processing imposed by TG or A23187 (Fig. 4k and l, Supplementary Fig. S5g and h).

Similar as IR, insulin-like growth factor-1 receptor (IGF1R) is also a substrate of FURIN [18], and its β subunit (IGF1Rβ) after FURIN processing was also decreased in SERCA2a^Thr484Ala^ and SERCA2a^+/−^ cardiomyocytes (Fig. 4a−d). Furthermore, TG and A23187 decreased IGF1Rβ but caused an accumulation of IGF1R precursor in NRVCs as well as in HEK293 cells (Fig. 4g and h, Supplementary Fig. S5c and d). TG- or A23187-elicited inhibition of IGF1R processing was relieved by overexpression of FURIN (Fig. 4k and l, Supplementary Fig. S5g and h).

The effect of the SERCA2a^Thr484Ala^ mutation on IRβ exhibited a tissue-dependent manner, and was manifested in the heart where SERCA2a is the dominant Ca^2+^ pump for SR Ca^2+^ re-uptake. In contrast, IRβ remained normal in skeletal muscle and white adipose tissue of the SERCA2a^Thr484Ala^ mice, in which SERCA1 and SERCA3 may dominate (Supplementary Fig. S2k−m).

Together, these data show that inhibition of SR Ca^2+^ re-uptake resulted from the SERCA2a^Thr484Ala^ mutation impairs precursor protein processing through down-regulation of FURIN in cardiomyocytes.

### Inhibition of SERCA2a promotes lysosomal degradation of FURIN

We then examined possible mechanisms underlying down-regulation of FURIN protein by inhibition of SERCA2a. Diminution of FURIN in SERCA2a^Thr484Ala^ and SERCA2a^+/−^ cardiomyocytes was most likely not due to *Furin* mRNA that remained normal in these cardiomyocytes (Supplementary Fig. S7a and b). Moreover, TG- or A23187-treatment did not affect *Furin* mRNA in HEK293 cells (Supplementary Fig. S7c and d). We suspected that protein stability might account for FURIN diminution induced by SERCA2a inhibition. Treatment with a proteosome inhibitor MG-132 did not affect TG- or A23187-elicited FURIN diminution (Fig. 4m and n, Supplementary Fig. S8a−d), suggesting that this FURIN diminution was not mediated by the proteosomes. In contrast, incubation of cardiomyocytes with a lysosome inhibitor bafilomycin-A1 prevented TG- or A23187-induced FURIN diminution (Fig. 4m and n, Supplementary Fig. S8a−d), suggesting that the lysosome-dependent degradation pathway may account for down-regulation of FURIN. Moreover, we found that a portion of FURIN was colocalized with the lysosomes in TG- or A23187-treated cardiomyocytes (Supplementary Fig. S8e). Together, these data suggest that FURIN diminution induced by SERCA2a inhibition was most likely mediated by protein degradation through the lysosomes.

### The SERCA2a^Thr484Ala^ knockin mice developed cardiomyopathy

**Figure 5:**
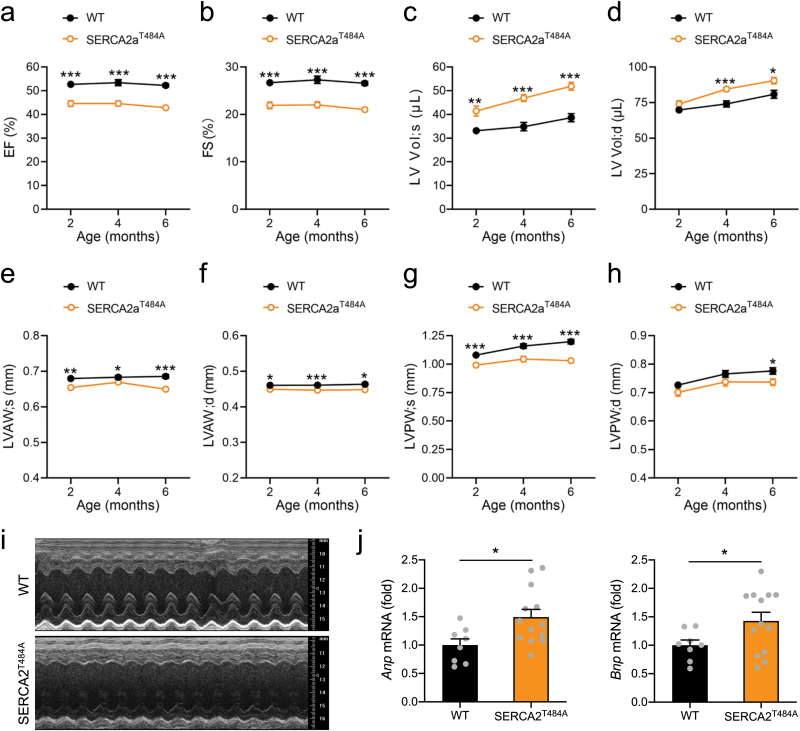
Cardiac function and remodeling of the SERCA2a^T484A^ knockin mice. (a−i) Echocardiography performed on the anaesthetized male SERCA2a^T484A^ knockin mice and WT littermates at the indicated ages. (a) EF. (b) FS. (c) Systolic LV Vol. (d) Diastolic LV Vol. (e) Systolic LVAW. (f) Diastolic LVAW. (g) Systolic LVPW. (h) Diastolic LVPW. (i) Representative images of echocardiography. *n* = 18–24. (j) Expression of *Anp* and *Bnp* mRNA in the heart of WT and SERCA2a^T484A^ knockin mice (4-month-old, male and female). *n* = 9–13. The data are given as the mean ± SEM. Statistical analyses were carried out using Student’s *t*-test. * indicates *P* < 0.05, **P < 0.01, and ***P < 0.001.

We next sought to find out how the SERCA2a^Thr484Ala^ knockin mutation impacts on cardiac function. Ejection fraction (EF) and fraction shortening (FS) became significantly lower in the male SERCA2a^Thr484Ala^ knockin mice than in the WT littermates from the age of 2 months (Fig. 5a, b and i). The LV volumes of these animals were larger than those of the WT littermates under both systolic and diastolic conditions (Fig. 5c, d and i). Both anterior and posterior walls were thinner in the SERCA2a^Thr484Ala^ heart than those in the WT heart under systolic as well as diastolic conditions (Fig. 5e−i). The SERCA2a^Thr484Ala^ knockin mutation exerted similar effects in female mice, and also caused cardiomyopathy from the age of 2 months (Supplementary Fig. S9a−h). These data show that the SERCA2a^Thr484Ala^ knockin mutation caused dilated cardiomyopathy in mice. Molecular analyses revealed that the SERCA2a^Thr484Ala^ knockin mice underwent remodeling in their hearts. A-type natriuretic peptide (ANP) and B-type natriuretic peptide (BNP) are cardiac neurohormones that are released from ventricles of dilated hearts [19], and their mRNA levels were significantly increased in the heart of SERCA2a^Thr484Ala^ knockin mice (Fig. 5j). The ATP levels were increased by more than 2-fold in the heart of SERCA2a^Thr484Ala^ knockin mice (Supplementary Fig. S2j), showing that the decline of cardiac function in these mice was not due to energy deficiency but rather due to impaired Ca^2+^ homeostasis.

These data demonstrate that SERCA2a-Thr^484^ phosphorylation is critical for cardiac function under normal feeding conditions.

### Decline of SERCA2a-Thr^484^ phosphorylation underlies the early pathogenesis of WD-induced DCM

**Figure 6:**
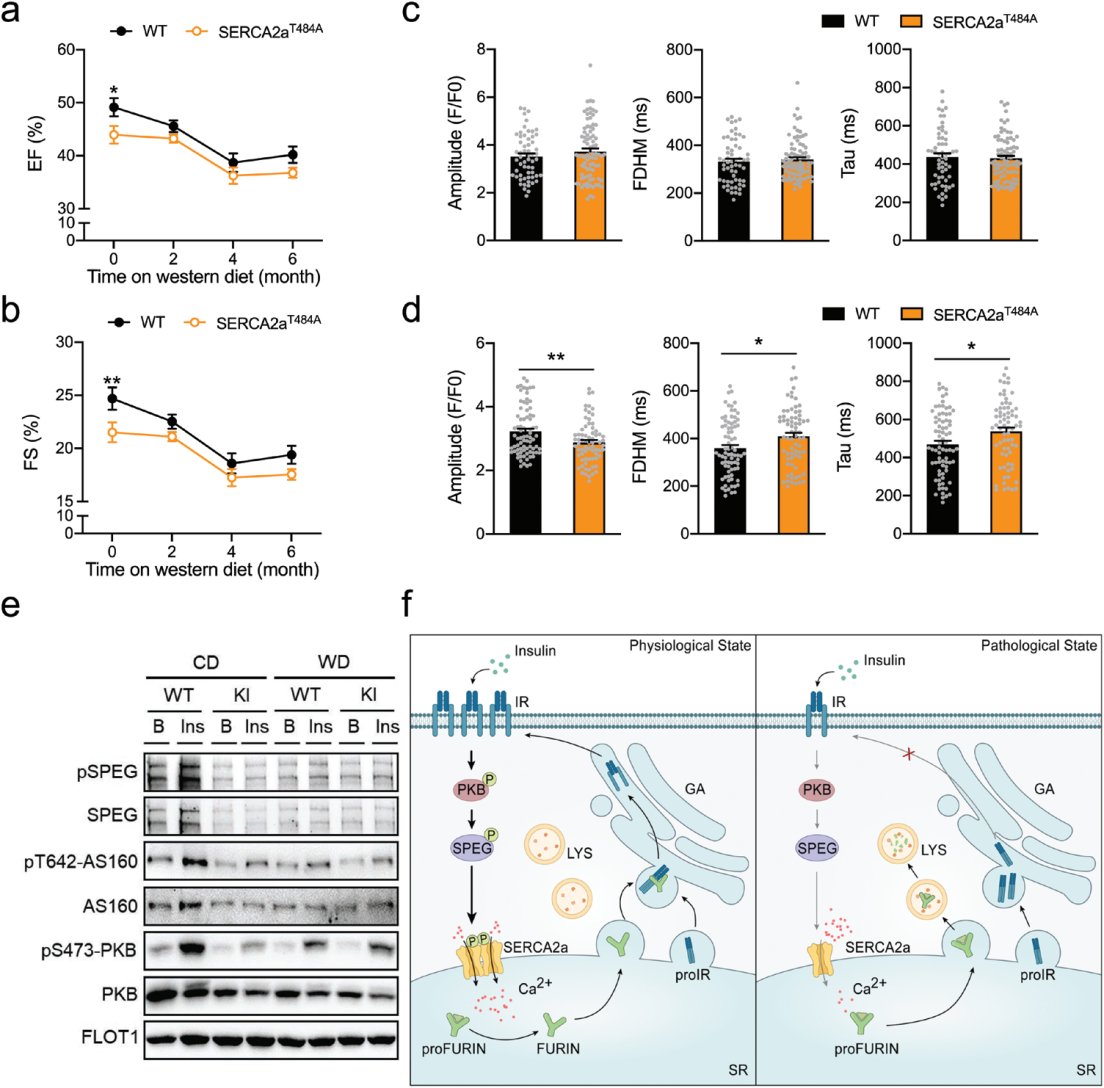
SERCA2a phosphorylation in the development of WD-induced DCM. (a and b) EF (a) and FS (b) measured via echocardiography in the male WT and SERCA2a^T484A^ mice fed with a WD for periods indicated in the figure. Statistical analyses were performed via Student’s *t*-test for each time point between the WT and SERCA2a^T484A^ mice. EF: * indicates *P* < 0.05 (WT/0 M vs SERCA2a^T484A^/0 M). FS: ** indicates *P* < 0.01 (WT/0 M vs SERCA2a^T484A^/0 M). *n* = 6−9. (c) Ca^2+^ transients elicited by electrical stimulation in primary cardiomyocytes isolated from the WT and SERCA2a^T484A^ male mice (ad libitum) fed with WD for 7 months (WD feeding started at the age of 4-month). Amplitudes, FDHM, and Tau of Ca^2+^ transients from 59 WT cells (2 mice) and 83 SERCA2a^T484A^ cells (2 mice), respectively. (d) Ca^2+^ transients elicited by electrical stimulation in primary cardiomyocytes isolated from the WT and SERCA2a^T484A^ mice (15-month-old, male, ad libitum) fed with CD. Amplitudes, FDHM, and Tau of Ca^2+^ transients from 78 WT cells (2 mice) and 70 SERCA2a^T484A^ cells (2 mice), respectively. (e) Phosphorylation and expression of SPEG, AS160, and PKB in primary cardiomyocytes isolated from the WT or SERCA2a^T484A^ mice (15-month-old, male, fasted overnight) fed with a CD or a WD in response to insulin (300 nM, 30 min). (f) A diagram representing the proposed model in which SERCA2a is a critical mediator of insulin action in the heart. Insulin-stimulated SERCA2a-Thr^484^ phosphorylation couples FURIN-dependent precursor protein processing with cardiac contraction through regulating SR Ca^2+^ re-uptake to maintain cardiac function. Impaired phosphorylation of SERCA2a-Thr^484^ inhibits SR Ca^2+^ re-uptake and promotes FURIN degradation through lysosomes, which results in impaired processing of IR precursor. Therefore, impaired phosphorylation of SERCA2a-Thr^484^ is both a cause and a consequence of cardiac insulin resistance, and underlies the early pathogenesis of DCM induced by WD. The data are given as the mean ± SEM. Statistical analyses were carried out using Student’s *t*-test. * indicates *P* < 0.05, and **P < 0.01.

We next sought to find out a potential role of SERCA2a-Thr^484^ phosphorylation in the pathogenesis of DCM. To this end, the SERCA2a^Thr484Ala^ knockin mice and their WT littermates were fed with WD for induction of DCM. As expected, WD feeding induced obesity and elevated fasting blood glucose levels in the WT mice (Supplementary Fig. S10a and b). The SERCA2a^Thr484Ala^ knockin mice did not differ from their WT littermates regarding their body weight gain on WD (Supplementary Fig. S10a). Their fasting blood glucose was increased to the levels similar to that in the WT mice (Supplementary Fig. S10b). Both WT and SERCA2a^Thr484Ala^ knockin mice developed glucose intolerance when fed with WD as compared to CD, and they displayed no difference in the clearance of blood glucose after administered with a bolus of glucose (Supplementary Fig. S10c and d). These data suggest that the SERCA2a^Thr484Ala^ knockin mutation may not affect whole-body glucose homeostasis in mice. As aforementioned, WD feeding elicited a progressive decline of cardiac function as evidenced by the EF and FS in the WT mice within the first 4 months (Fig. 6a and b). The EF and FS displayed no further decline from 4 to 6 months on WD feeding (Fig. 6a and b). The SERCA2a^Thr484Ala^ knockin mice had lower EF and FS than their WT littermates before fed with WD (Fig. 6a and b). Interestingly, their EF and FS did not change within the first 2 months on WD feeding. Afterwards, these two parameters started to decline in the SERCA2a^Thr484Ala^ knockin mice till 4 months when they reached a plateau (Fig. 6a and b).

We measured Ca^2+^ transients in primary cardiomyocytes isolated from mice at a late stage of WD feeding. The peaks, FDHM and Tau of Ca^2+^ transients did not differ between the two genotypes when cardiomyocytes were isolated from the WD-fed mice (Fig. 6c). In contrast, Ca^2+^ transients of the SERCA2a^Thr484Ala^ knockin cardiomyocytes had smaller peaks and larger FDHM and Tau than those of the WT cardiomyocytes when cells were isolated from the CD-fed mice (Fig. 6d). Again, we observed that the SERCA2a^Thr484Ala^ knockin mutation caused insulin resistance in cardiomyocytes isolated from the CD-fed mice. The WD feeding caused insulin resistance in WT cardiomyocytes to a level similar to the SERCA2a^Thr484Ala^ cardiomyocytes from the CD-fed or WD-fed mice (Fig. 6e).

Together, these data demonstrate that impairment of SERCA2a-Thr^484^ phosphorylation underlies the early pathogenesis of DCM induced by WD.

## Discussion

Our findings demonstrate that SERCA2a is critical for mediating insulin action in the heart, and that its Thr^484^ phosphorylation couples precursor protein processing with cardiac contraction through regulating SR Ca^2+^ re-uptake to maintain cardiac function (Fig. 6f). Impaired phosphorylation of SERCA2a-Thr^484^ is both a cause and a consequence of insulin resistance in cardiomyocytes, which underlies the early pathogenesis of DCM induced by WD.

Insulin elicits activation of the PI3K−PKB pathway through its receptor to exert its physiological effects. These proximal components of insulin signaling are present in all insulin-responsive tissues. However, it has long been recognized that some tissues may develop selective insulin resistance [20, 21]. For instance, in the insulin resistant liver, insulin is no longer able to inhibit hepatic gluconeogenesis but exhibits enhanced activities to promote lipogenesis [22]. The existence of such selective insulin resistance leads to a recent proposal of tissue- and/or pathway-specific insulin action [23]. Certain tissue/pathway-specific distal components of insulin signaling or effectors are likely to be involved in execution of such a selective insulin action. In agreement with this notion, the SPEG-SERCA2a signal module at the downstream of insulin pathway is specifically present in striated muscle, and mediates insulin action in the heart. This signal module not only exhibits tissue-specific distribution but also generates pathway-selective output of insulin action. It regulates Ca^2+^ homeostasis in the heart but has no effect on whole-body glucose metabolism. Therefore, disruption of this signal module via introduction of either SPEG^3A^ mutation [11] or SERCA2a^Thr484Ala^ mutation (this study) causes selective insulin resistance in the heart, impairs Ca^2+^ homeostasis, and results in cardiomyopathy.

Our data show that rhythmic cardiac contraction that drives the pumping action of the heart is intrinsically coupled with FURIN-dependent precursor protein processing through Ca^2+^ shuttling between the cytosol and SR. It is known that Ca^2+^ regulates FURIN maturation and enzymatic activity [17, 24]. Our findings reveal another regulatory mechanism of Ca^2+^ in control of FURIN, in which Ca^2+^ diminution in SR promotes lysosome-mediated degradation of FURIN. As a proprotein convertase, FURIN has a variety of substrates including IR whose signaling leads to SPEG-dependent Thr^484^ phosphorylation of SERCA2a [11, 25]. Thus, IR, PKB, SPEG, SERCA2a, and FURIN form a circuit regulating insulin sensitivity and cardiac function. Cardiac insulin resistance decreases the activity of PKB-SPEG axis, which results in hypo-phosphorylation of SERCA2a-Thr^484^ and consequent abnormal cellular Ca^2+^ handling. In turn, impaired Ca^2+^ homeostasis aggravates cardiac insulin resistance through impairing FURIN-dependent processing of IR precursor. Therefore, disruption or disturbance of different nodes within this regulatory circuit may impair cellular Ca^2+^ handling and cause cardiac insulin resistance, which eventually leads to the development of cardiomyopathy.

Though discovered about four decades ago, the pathogenesis of DCM is still not well understood. Physiological homeostasis of glucose, lipids and Ca^2+^ is impaired in diabetic hearts together with oxidative stress, inflammation, and mitochondrial dysfunction, which may contribute to the pathogenesis of DCM [3, 5]. The relative contributions of these alterations to the development of DCM need to be defined particularly in a temporal context. The SERCA2a^Thr484Ala^ knockin mice allow us to address this question with respect to possible roles of impaired Ca^2+^ homeostasis. Our data with these SERCA2a^Thr484Ala^ mice demonstrate the importance of SERCA2a-Thr^484^ phosphorylation in maintenance of cardiac function, which is in agreement with previous studies associating cardiac dysfunction of diabetic hearts with impaired Ca^2+^ handling through SERCA2a in cardiomyocytes [5]. Importantly, our findings using this SERCA2a^Thr484Ala^ model reveal distinct phases in the pathogenesis of DCM. Impaired SERCA2a-Thr^484^ phosphorylation and Ca^2+^ homeostasis due to insulin resistance underlie the early pathogenesis of DCM. Based on this observation, we propose a “tandem hit” hypothesis for the pathogenesis of DCM, in which impaired Ca^2+^ homeostasis due to insulin resistance is the major insult in an early phase of DCM. Cardiac function continuously declines in a middle phase until it reaches a plateau in a late phase. Neither causal molecules nor characteristic changes are clear for the middle and late phases of DCM, which deserves investigations in future. Identification of molecular markers for each phase of DCM is of clinical importance for diagnosis. Our data suggest that abnormal Ca^2+^ handling alone might not be sufficient to elicit an early onset of the middle phase of DCM in the SERCA2a^Thr484Ala^ knockin mice. Some unknown factors might be required for the onset of the middle phase, whose accumulation might take time in similar rates in the SERCA2a^Thr484Ala^ knockin mice and their WT littermates upon WD feeding. Alternatively, we might have missed the onset point of the middle phase due to the low time resolution of our study, which might be earlier in the SERCA2a^Thr484Ala^ knockin mice than in the WT littermates. Further studies are required to address these open questions in the future.

Restoration of SERCA2a activity through posttranslational modifications has attracted attentions for development of therapeutic strategies to treat heart failure. For instance, SUMOylation of SERCA2a is important for its activity [26], which can be enhanced through gene transfer of SUMO-1 or small molecule activator [27, 28]. Increased SUMOylation of SERCA2a through these experimental medicines prevents heart failure [27, 28]. Our findings in this study show that hypo-phosphorylation of SERCA2a underlies the early pathogenesis of DCM. Therefore, restoration of SERCA2a-Thr^484^ phosphorylation might be of clinical value to treat DCM. SPEG is a PKB substrate as well as an upstream kinase for SERCA2a [10, 11]. In insulin resistant hearts, insulin fails to activate the SK2 domain of SPEG, resulting in hypo-phosphorylation of SERCA2a. The SK2 domain of SPEG on its own is capable of phosphorylating SERCA2a-Thr^484^ in cardiomyocytes [10]. It is therefore intriguing to find out whether expression of SK2 of SPEG might be a possible way to treat DCM in its early phase.

In summary, we showed that SERCA2a-Thr^484^ phosphorylation couples precursor protein processing with cardiac contraction through regulating SR Ca^2+^ re-uptake and is critical for cardiac function. Impaired phosphorylation of SERCA2a-Thr^484^ due to insulin resistance underlies the early pathogenesis of DCM. Our findings have therapeutic implications for treatment of DCM.

## Materials and methods

### Materials

Recombinant human insulin was bought from Novo Nordisk (Bagsvaerd, Denmark). TG was bought from Sigma-Aldrich (Cat. No. T9033), and A23187 from Calbiochem (CAS 52665-69-7). Protein G-Sepharose was purchased from GE Healthcare (Buckinghamshire, UK). WD (Cat. No. D12079B) was purchased from Research Diets (USA). All other chemicals were bought from Sigma-Aldrich (Shanghai, China) or Sangon Biotech (Shanghai, China).

### Antibodies

The rabbit antibody against SPEG (Cat No. 12472-RP01) was bought from Sino Biologicals (Beijing, China). The total SERCA2a antibody (Cat No. 13985-1-AP), total FURIN antibody (Cat No. 18413-AP), and GAPDH (Cat No. 60004-1-Ig) antibody were from Proteintech (Wuhan, China). The total PLB (Cat No. A17964) and pS16-PLB (Cat No. AP0907) were from ABclonal (Wuhan, China). The pT484-SERCA2a antibody was as previously reported [10] (PMID: 30566039). The antibodies that recognize PAS (Cat No. 9611), pS473-PKB (Cat No. 9271), total PKB (Cat No. 9272), pT642-AS160 (Cat No. 8881), total AS160 (Cat No. 2670), pS21/9-GSK3 (Cat No. 8566), total GSK3 (Cat No. 5676), and total IGF1Rβ (Cat No.9750) were from Cell Signaling Technology. The antibodies that recognize total IRβ (Cat No. sc-711) and total Flotillin1 (Cat No. sc-25506) were from Santa Cruz (Dallas, TX). The antibodies that recognize LAMP1 (Cat No. ab25245), GLUT1 (Cat No. ab652), GLUT4 (Cat No. ab654), and CD36 (Cat No. ab133625) were bought from Abcam (Cambridge, UK). The antibody against RyR2 (Cat No. BS72313) was bought from Bioworld (Bloomington, MN). The antibody that recognizes pS2808-RyR2 (Cat No. A010-30AP) was bought from Badrilla (Leeds, UK).

### Molecular biology

Human SERCA2a (NP_001672.1) and human FURIN (NP_001276752.1) cDNAs were cloned into pcDNA5-FRT/TO vectors with tags for expression in mammalian cells. All plasmids were sequenced at Life Technologies (Shanghai, China).

### Generation of SERCA2a^T484A^ knockin and SERCA2a knockout mice

Generation of SERCA2a^T484A^ knockin and SERCA2a knockout mice on a C57Bl/6J background was carried out by the transgenic facility at Nanjing University using the CRISPR/Cas9-based strategy outlined in Fig. 2a and Supplementary Fig. S4a. The Thr^484^ residue (the surrounding sequence is QLMKKEF**t**LEF, Thr^484^ shown in lower case bold) on SERCA2a was changed to alanine via CRISPR/Cas9-assisted knockin substitution to generate SERCA2a^T484A^ knockin mice. An *Hpy8i* enzyme restriction site in WT allele was removed through synonymous mutations to facilitate genotyping. The mutated region (271 bp) was amplified using two primers (5ʹ-AGGAAAGCCTCAGTCTGCAC-3ʹ and 5ʹ-GGCTGCCAAAAAGACCAGTG -3ʹ), and digested with *Hpy8i* to generate 123/148 bp cleaved products for WTs and 271 bp noncleaved products for SERCA2a^T484A^ knockins.

For the generation of SERCA2a knockout mice, the region between 2043 and 2052 nt of SERCA2a (NM_009722.3) was deleted, which resulted in a frameshift mutation. Mouse genotype was identified through DNA sequencing.

### Mouse breeding and husbandry

The Ethics Committee at Model Animal Research Center of Nanjing University reviewed and approved all animal protocols used in this study. Mice were produced and maintained in an animal facility that is free of specific pathogens and has a light/dark cycle of 12 h. Mice have free access to food and water unless otherwise stated.

Heterozygote × heterozygote breeding pairs were set up to generate SERCA2a^T484A^ homozygotes and their WT littermates. Heterozygote × WT breeding pairs were used to produce SERCA2a-KO heterozygotes and their WT littermate controls.

### Oral glucose tolerance test

Oral glucose tolerance test was performed in mice after deprivation of food overnight (16 h). Mice were orally administered with a bolus of glucose (1.5 mg/g), and then tail bled at indicated time intervals for measurement of blood glucose using a Contour-TS glucometer (Bayer).

### ATP assay

ATP contents in the heart were measured using a luciferase–luciferin ATP Assay Kit (Beyotime, China) following the manufacturer’s instructions.

### Tissue lysis

Mouse tissues were harvested, snap-frozen in liquid nitrogen, and stored at −80°C before lysis. Frozen tissues were homogenized in ice-cold lysis buffer with a Polytron homogenizer (Kinematica, Luzern, Switzerland). After further incubation on ice for 30 min, homogenates were centrifuged to remove tissue debris. Protein concentrations of tissue lysates were measured using Bradford reagent (Thermo Scientific).

### Cell culture and transfection

Human embryonic kidney HEK293 cells were obtained from the Cell Resource Center, Chinese Academy of Medical Sciences and Peking Union Medical College (China), and maintained in Dulbecco’s Modified Eagle Medium (DMEM) containing 10% (v/v) fetal bovine serum. Cells were examined for mycoplasma contamination on a regular basis. Cell transfection with plasmid DNA was carried out via a PEI-mediated method. Cells were lysed 2 days after transfection, and protein concentrations of cell lysates were determined using Bradford reagent (Thermo Scientific).

### Immunoblotting

Protein extracts or immunoprecipitates were eletrophoretically separated via SDS-PAGE, and transferred onto nitrocellulose membranes via immunoblotting. After blocked with 5% milk, membranes were incubated with primary antibodies overnight at 4°C. Membranes were washed for three times, and further incubated with horseradish-peroxidase-conjugated secondary antibodies for 1 h at room temperature. After removal of unbound secondary antibodies through intensive wash, membranes were incubated with ECL substrates (GE Healthcare, UK), and chemiluminescence signals were detected using a gel documentation system (Tanon, China). Quantitation of immunoblots was carried out using ImageJ.

### IR processing assay

HEK293 cells transfected with Flag-IR for 48 h were treated with or without 2 μM TG for 90 min. During this period, nascent proteins were labeled with Click-IT^TM^ AHA (l-azidohomoalaine) from Thermo Fisher Scientific for indicated time. After labeling, total Flag-IR was immunoprecipitated from cell lysates and reacted with Click-IT^TM^ Protein Reaction Buffer Kit. The nascent Flag-IR (precursor) and mature IRβ were detected in the immunoprecipitates via immunoblotting using the HRP-labeled Avidin antibody. Immunoblotting signals were quantified using ImageJ, and IR processing was defined as the ratio of mature IRβ to IR precursor.

### Colocalization assay in NRVCs

NRVCs were treated with or without A23187 (1 μM)/TG (2 μM) for 4 h. After fixation and permeabilization, cells were stained with LAMP1 and FURIN antibody, and then incubated with Cy3- and FITC-conjugated secondary antibodies, respectively. Images were taken using a DeltaVision Elite imaging apparatus (GE Healthcare).

### Echocardiography

Echocardiography (Echo) analysis was carried out as previously described [29]. Briefly, mice were anaesthetized with gaseous isoflurane, and then subjected to Echo analysis using a Vevo 770 high-resolution *in vivo* micro-imaging system (VisualSonics, Inc.). M-mode images were collected through positioning a 30 MHz RMV-707B ultrasonic probe at a 90° angle between the probe and the heart, and used to measure left ventricle anterior wall (LVAW), left ventricle posterior wall (LVPW), left ventricle internal dimension (LVID), and left ventricle volume (LV Vol) of systole and diastole. EF and FS are calculated using the following equations, EF% = [(LV Vol;d − LV Vol;s)/LV Vol;d] × 100% and FS% = [(LVID;d − LVID;s)/LVID;d] × 100%.

### Isolation of primary cardiomyocytes

Primary cardiomyocytes were isolated from adult mice as previously described [30]. Briefly, mouse heart was mounted onto a catheter after rapid removal from heparin-treated animals, and perfused with a collagenase solution (1 mg/ml) using a Langendorff system (ADInstruments). Cell suspension was passed through a 100 μm filter, and resultant primary cardiomyocytes were gently washed in Krebs–Henseleit buffer B containing 5 mM taurine and 10 mM 2,3-butanedione monoximine for three times. Step-wise increases of Ca^2+^ were supplemented in the wash buffer (0.1 mM for the first round, 0.2 mM for the second round, and 0.6 mM for the third round).

Isolation of neonatal rat cardiomyocytes was carried out as previously described [31]. Briefly, ventricles of neonatal animals (postnatal day 0−3, rat strain Sprague Dawley) were chopped into cubes (~1 mm) that were subsequently incubated with 0.25% trypsin at 4°C overnight, followed by digestion with collagenase (1 mg/ml) at 37°C for 15 min. After filtering through a 200 μm mesh, resultant cells were plated in DMEM containing 10% (v/v) fetal bovine serum for 1 h to remove fibroblasts. After removal of fibroblasts, cardiomyocytes were re-seeded in fresh DMEM plus 10% (v/v) fetal bovine serum, and used for treatments with indicated chemicals.

### Ca^2+^ imaging in primary cardiomyocytes

For assay of Ca^2+^ sparks, Ca^2+^-loaded primary cardiomyocytes were incubated with 5 μM Fluo-4-AM (Thermo Fisher Scientific) and imaged using a Zeiss LSM880 confocal microscope. For Ca^2+^ transient assay, primary cardiomyocytes in Hanks buffer containing 1 mM MgCl_2_, 1 mM CaCl_2,_ and 2% (w/v) BSA were loaded with 5 μM Fluo-4-AM (Thermo Fisher Scientific), and then stimulated with a GRASS S48 stimulator (frequency 0.5 Hz, duration 60 ms, decay 40 ms, voltage 80 V, and repeat). Ca^2+^ images were taken using a Zeiss LSM880 confocal microscope that was set in a line-scan mode, and analyzed using IDL5.5 (Harris Geospatial Solutions). The decay time Tau was defined as the period lasting from the peak of calcium transients to 63% from the peak to the basal level in the fading phase.

### Measurement of SR Ca^2+^ load

For measurement of SR Ca^2+^ load, cardiomyocytes were paced with electrical stimulation at 0.5 Hz until twitch characteristics became stabilized before each caffeine application. Afterwards, caffeine (10 mM) was rapidly applied to fully paced cardiomyocytes to induce release of Ca^2+^ from SR. The amplitude of the caffeine-induced Ca^2+^ transient was used as an index of SR Ca^2+^ content.

### RNA isolation and quantitative PCR

Total RNA was extracted from tissues or cells using the TRIzol® Reagent (Life Technologies), and used to generate cDNAs via reverse-transcription using a PrimeScript® RT reagent kit (DRR047A, TaKaRa). Resultant cDNAs were used to determine gene expression through quantitative PCR using an Applied Biosystems® StepOnePlus^TM^ Real-Time PCR system (Life Technologies) with the primers listed in Supplementary Table 1.

### Statistical analysis

Data were analyzed using Prism software (GraphPad, San Diego, CA). Comparisons of two groups and multiple groups were carried out via *t*-test or two-way ANOVA, respectively. Statistical significance was considered for differences at *P* < 0.05.

## Supplementary Material

loac013_suppl_Supplementary_Figures
