# Supplementary material for: Impaired SERCA2a phosphorylation causes diabetic cardiomyopathy through impinging on cardiac contractility and precursor protein processing
Source: Life Metab. 2022 Jul 28;1(1):54–66. doi: 10.1093/lifemeta/loac013 (PMC11749685; doi:10.1093/lifemeta/loac013)

Impaired SERCA2a phosphorylation causes diabetic cardiomyopathy through impinging on cardiac contractility and precursor protein processing

Chao Quan, Sangsang Zhu, Ruizhen Wang, Jiamou Chen, Qiaoli Chen, Min Li, Shu Su, Qian Du, Minjun Liu, Hong-Yu Wang* and Shuai Chen*

Supplementary Table S1 Primers for real-time PCR

Gene name	Species	Forward primer	Reverse primer
Furin	Human	5′-CCTTCTTCCGTGGGGTTAG-3′	5′-GCAGTTGCAGCTGTCATGTT-3′
Gapdh	Human	5′-GGAGCGAGATCCCTCCAAAAT-3′	5′-GGCTGTTGTCATACTTCTCATGG-3′
Furin	Mouse	5′-TAGCAGGCAATTATGACCCTGG-3′	5′-TAAGCTACACCTACGCCACAG-3′
Anp	Mouse	5′-TCGTCTTGGCCTTTTGGCT-3′	5′-TCCAGGTGGTCTAGCAGGTTCT-3′
Bnp	Mouse	5′-AAGCTGCTGGAGCTGATAAGA-3′	5′-GTTACAGCCCAAACGACTGAC-3′
Col3a1	Mouse	5′-CCCAGAACATTACATACCA-3′	5′-GATTAAAACAAGATGAACAC-3′
Rcan1.4	Mouse	5′-GTGTGGCAAACGATGATGTC-3′	5′-AGGAACTCGGTCTTGTGCAG-3′
Glut1	Mouse	5′-ATGAAAGAAGAGGGTCGGCA-3′	5′-TCCAGCTCGCTCTACAACAA-3′
Glut4	Mouse	5′-GCCATCGTCATTGGCATTCT-3′	5′-CGCTTTAGACTCTTTCGGGC-3′
Hk2	Mouse	5′-CCCTGTGAAGATGTTGCCCACT-3′	5′-CCTTCGCTTGCCATTACGCACG-3′
Pfk2	Mouse	5′-CCTATGCACTAGCCAACTTC-3′	5′-CACCCGCATCAATCTCATTC-3′
Lpl	Mouse	5′-TTCAACCACAGCAGCAAGAC-3′	5′-CTGGATAATGTTGCTGGGCC-3′
Cd36	Mouse	5′-GAATGGGCTGTGATCGGAAC-3′	5′-ACGTCATCTGGGTTTTGCAC-3′
Fatp1	Mouse	5′-CTACCACTCTGCAGGGAACA-3′	5′-CAGGTAGCGGCAGATTTCAC-3′
Fabp3	Mouse	5′-CCCCTCAGCTCAGCACCAT-3′	5′-CAGAAAAAATCCCAACCCAAGAAT-3′
Atgl	Mouse	5′-CAACGCCACTCACATCTACG-3′	5′-ACCAGGTTGAAGGAGGGATG-3′
Cpt1	Mouse	5′-ACTCCTGGAAGAAGAAGTTCA-3′	5′-AGTATCTTTGACAGCTGGGAC-3′
Gapdh	Mouse	5′-AGGTCGGTGTGAACGGATTTG-3′	5′-TGTAGACCATGTAGTTGAGGTCA-3′
36B4/Rplp0	Mouse	5′-TAAAGACTGGAGACAAGGTG-3′	5′-GTGTACTCAGTCTCCACAGA-3′

Supplemetary Figure Legends

Supplementary Figure S1 Echocardiographic parameters of mice fed with CD or WD

a. Quantification of IRβ in primary cardiomyocytes isolated from CD- or WD-fed male mice (10-month-old, fasted overnight). The mice were fed with WD for 32 weeks. Blots were shown in Fig. 1c. Signals of

IRβ were normalized with FLOT1. n = 6.

b-e. Quantification of phosphorylation of SERCA2a (b), SPEG (c), AS160 (d) and PKB (e) in primary cardiomyocytes isolated from CD- or WD-fed male mice (10-month-old, fasted overnight). The mice were fed with WD for 32 weeks. Primary cardiomyocytes were stimulated with or without insulin (300 nM, 30 min) before lysed for immunoblotting analyses. Blots were shown in Fig. 1c. Signals of phosphorylation of SERCA2a, SPEG, AS160 and PKB were normalized with their respective total protein. n = 3.

f. Echocardiography was performed on the anaesthetized male C57/BL6J male mice fed with CD or WD (WD feeding started at the age of 2-month). Systolic left ventricle volume (LV Vol; s), diastolic left ventricle volume (LV Vol; d), systolic left ventricle anterior wall (LVAW; s), diastolic left ventricle anterior wall (LVAW; d), systolic left ventricle posterior wall (LVPW; s), and diastolic left ventricle posterior wall (LVPW; d) were measured via echocardiography. CD, chow diet; WD, western diet. n = 6-12.

The data are given as the mean ± SEM. Statistical analyses were carried out via two-way ANOVA for b-e, and via t-test for a and f. * indicates $p < 0.05$, ** $p < 0.01$, and *** $p < 0.001$.

Supplementary Figure S2 Generation and basic characterization of the SERCA2a^{T484A} knockin mice

a. Genomic DNA sequences of the WT and SERCA2a^{T484A} allele in the heterozygous SERCA2a^{T484A} mice.

b. Genotyping of the WT and SERCA2a^{T484A} mice via PCR.

c-d. Expression of SERCA2a, SPEG, GLUT1, GLUT4 and CD36 in the heart of WT and SERCA2a^{T484A} mice (2-month-old, female). c, representative blots. d, quantitative data. n = 5-6.

e-f. Fasting blood glucose in male WT and SERCA2a^{T484A} mice at the age of 4-month (e, n = 7-8) and 9-month (f, n = 5-8).

g. Glucose tolerance test in male WT and SERCA2a^{T484A} mice (4-month-old). The values show area under the curve during glucose tolerance test. AUC, area under the curve. n = 7-8.

h. Glucose tolerance test in male WT and SERCA2a^{T484A} mice (9-month-old). The values show area under the curve during glucose tolerance test. AUC, area under the curve. n = 5-8.

i. mRNA levels of glucose and lipid metabolic genes in the heart of WT and SERCA2a^{T484A} mice (4-month-old, male and female). n = 5-7.

j. ATP contents in the heart of WT and SERCA2a^{T484A} male mice (6-month-old). n = 6.

k-m. IRβ levels in skeletal muscle and WAT of WT and SERCA2a^{T484A} female mice (4-month-old). Blots shown in k were quantified in l (skeletal muscle) and m (WAT).

The data are given as the mean ± SEM. Statistical analyses were carried out using t-test. * indicates $p < 0.05$.

Supplementary Figure S3 Ca^{2+} homeostasis in the heart of SERCA2a^{T484A} mice

a-c. Ca^{2+} transients elicited by electrical stimulation (3 Hz) in primary cardiomyocytes isolated from the WT and SERCA2a^{T484A} female (4-month-old, fed ad libitum). Amplitude (a), full duration at half maximum (FDHM)(b) and time constant Tau (c) of Ca^{2+} transients were analyzed from 83 WT cells (6 mice) and 97 SERCA2a^{T484A} cells (6 mice).

d-f. Ca^{2+} transients elicited by electrical stimulation (0.5 Hz) in primary cardiomyocytes isolated from the WT and SERCA2a^{T484A} female (4-month-old, fed ad libitum). Amplitude (d), FDHM (e) and Tau (f) of Ca^{2+} transients were analyzed from 71 WT cells (6 mice) and 51 SERCA2a^{T484A} cells (6 mice).

g. mRNA levels of *Serca2a*, *Plb* and *Ryr2* in the heart of WT and SERCA2a^{T484A} mice (4-month-old, male and female). n = 5-7.

h-m. Phosphorylation and expression of PLB and RyR2 in the heart of female WT and SERCA2a^{T484A} knockin mice (2-month-old) were examined via immunoblotting. h, representative blots. i-m, quantitative data. n = 5-6.

The data are given as the mean ± SEM. Statistical analyses were carried out using t-test. * indicates $p < 0.05$, ** $p < 0.01$, and *** $p < 0.001$.

Supplementary Figure S4 Generation and basic characterization of the SERCA2a$^{+/-}$ mice

a. The diagrams show the WT and knockout allele of SERCA2a. For generation of SERCA2a knockout mice, the region between 2043~2052 nt of SERCA2a (NM_009722.3) were deleted through CRISPR/Cas9-mediated gene editing, which resulted in a frameshift mutation in *Serca2a*.

b. Genomic DNA sequences of the WT and SERCA2a-KO allele.

c. *Serca2a* mRNA levels in cardiomyocytes of the WT and SERCA2a$^{+/-}$ mice (3-month-old, female) via QPCR. n = 5.

d-e. Protein expression of SERCA2a in cardiomyocytes of the WT and SERCA2a$^{+/-}$ mice (3-month-old, male). d, immunoblots. e, quantification of SERCA2a immunoblotting signals. n = 6.

The data are given as the mean ± SEM. Statistical analyses were carried out using t-test. *** indicates $p < 0.001$.

Supplementary Figure S5 Insulin signaling in cells stimulated with A23187

a. PAS-reactive phosphorylation and phosphorylation of GSK3 and PKB in control or A23187 pretreated NRVCs stimulated with or without insulin. NRVCs were pretreated with or without 1 μM A23187 for 24h, and then stimulated with or without insulin (300 nM) for 30 min.

b. PAS-reactive phosphorylation and phosphorylation of PKB in control or A23187 pretreated H9C2 cardiomyocytes stimulated with or without insulin. H9C2 cardiomyocytes were differentiated for 5 days and pretreated with or without 1 μM A23187 for 24h. Cells were then stimulated with or without insulin (300 nM) for 30 min.

c-d. Expression of FURIN, precursor IR, IRβ, precursor IGF1R and IGF1Rβ in control or A23187-treated NRVCs. NRVCs were incubated with or without 1 μM A23187 for 24h. c, immunoblots. d, quantitative data. n = 6.

e-f. FURIN, precursor IR and IRβ protein levels in HEK293 cells treated with 1 μM A23187 for periods indicated in the figure. e, representative immunoblots. f, quantitative data. n = 4.

g-h. Effects of FURIN expression on IR and IGF1R processing in A23187-treated HEK293 cells. FURIN-mCherry or free mCherry was expressed in HEK293 cells. 24 hours after transfection, cells were treated with or without 1 μM A23187 for 24h. FURIN, precursor IR, IRβ, precursor IGF1R and IGF1Rβ protein levels were detected in cell lysates. g, immunoblots. h, quantitative data. n=3. † indicates $p < 0.001$ (A23187 vector vs Control vector) for precursor IR and IGF1R, $p < 0.01$ (A23187 vector vs Control vector) for IRβ, and $p < 0.05$ (A23187 vector vs Control vector) IGF1Rβ. ‡ indicates $p < 0.05$ (A23187 FURIN-mCherry vs Control FURIN-mCherry) for precursor IR, and $p < 0.01$ (A23187 FURIN-mCherry vs Control FURIN-mCherry) for precursor IGF1R.

The data are given as the mean ± SEM. Statistical analyses were carried out via t-test for d, via one-way ANOVA for f, and via two-way ANOVA for h. * indicates $p < 0.05$, ** $p < 0.01$, and *** $p < 0.001$.

Supplementary Figure S6 Protein expression of FURIN, precursor IR, and IRβ in HEK293 cells stimulated with TG

a-b. Expression of FURIN, precursor IR and IRβ proteins in HEK293 cells treated with TG (2 μM) for periods indicated in the figure. a, representative blots. b, quantitative data. n = 4.

The data are given as the mean ± SEM. Statistical analyses were carried out using one-way ANOVA. ** indicates $p < 0.01$, and *** $p < 0.001$.

Supplementary Figure S7 *Furin* mRNA in primary cardiomyocytes and HEK293 cells

a. *Furin* mRNA levels in primary cardiomyocytes from the WT and SERCA2a^{T484A} male mice (2-month-old). n = 6.

b. *Furin* mRNA levels in primary cardiomyocytes from the WT and SERCA2a$^{+/-}$ female mice (3-month-old). n = 6.

c. *Furin* mRNA levels in HEK293 cells treated with thapsigargin (2 μM) for periods indicated in the figure. n = 6.

d. *Furin* mRNA levels in HEK293 cells treated with A23187 (1 μM) for periods indicated in the figure. n = 6.

The data are given as the mean ± SEM. Statistical analyses were carried out using t-test.

Supplementary Figure S8 Lysosomal degradation of FURIN in A23187- or TG-treated cardiomyocytes

a-b, FURIN expression level in HEK293 cells that were treated with A23187 (1 μM) for 4 hours in the absence or presence of bafilomycin A1 (400 nM) or MG132 (10 μM). a, immunoblots. b, quantitative data. n=3.

c-d. FURIN expression level in NRVCs that were treated with A23187 (1 μM) for 4 hours in the absence or presence of bafilomycin A1 (400 nM) or MG132 (10 μM). c, immunoblots. d, quantitative data. n=3.

e. Colocalization of FURIN with the lysosome in control and TG (2 μM) or A23187 (1 μM) treated NRVCs. Bars indicate 10 μM in length.

The data are given as the mean ± SEM. Statistical analyses were carried out via one-way ANOVA. * indicates $p < 0.05$, and *** $p < 0.001$.

Supplementary Figure S9 Cardiac function of the female SERCA2a^{T484A} knockin

mice

Echocardiography was performed on the anaesthetized female WT and SERCA2a^{T484A} knockin mice and wild-type littermates at the indicated ages to measure ejection fraction (a), fraction shortening (b), systolic left ventricle volume (c), diastolic left ventricle volume (d), systolic left ventricle anterior wall (e), diastolic left ventricle anterior wall (f), systolic left ventricle posterior wall (g), and diastolic left ventricle posterior wall (h). n = 8-13.

The data are given as the mean ± SEM. Statistical analyses were carried out using t-test. * indicates $p < 0.05$, ** $p < 0.01$, and *** $p < 0.001$.

Supplementary Figure S10 Blood glucose and body weight in WT and SERCA2a^{T484A} knockin mice fed with CD or WD

a. Body weights of the male WT and SERCA2a^{T484A} knockin mice (9-month-old) fed with WD for 20 weeks. n = 9-15.

b. Blood glucose levels after 4 hours fast in male WT and SERCA2a^{T484A} knockin mice (8-month-old) that were fed with WD for 16 weeks. n = 6-8.

c. Oral glucose tolerance test in male WT and SERCA2a^{T484A} mice (8-month-old) that were fed with WD for 16 weeks. n = 6-8.

d. The values show area under the curve (AUC) during glucose tolerance test.

The data are given as the mean ± SEM. Statistical analyses were carried out via two-way ANOVA. * indicates $p < 0.05$, and *** $p < 0.001$.

Supplementary Figure S1

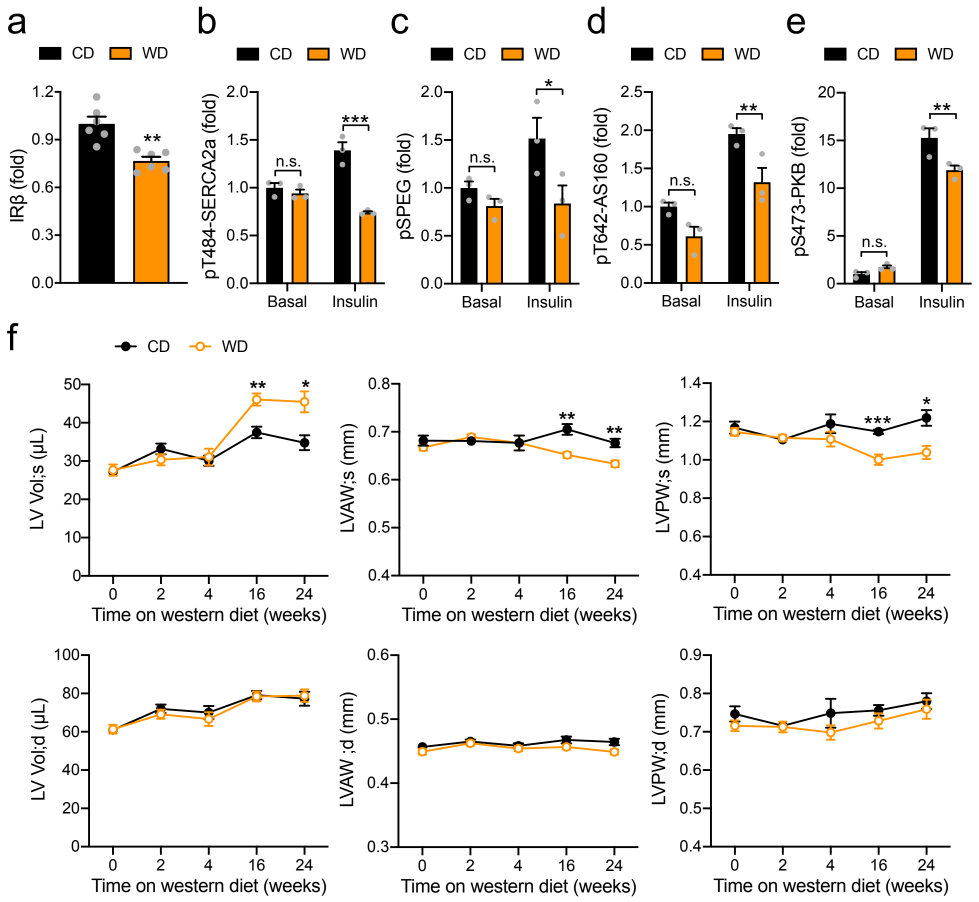

Supplementary Figure S2

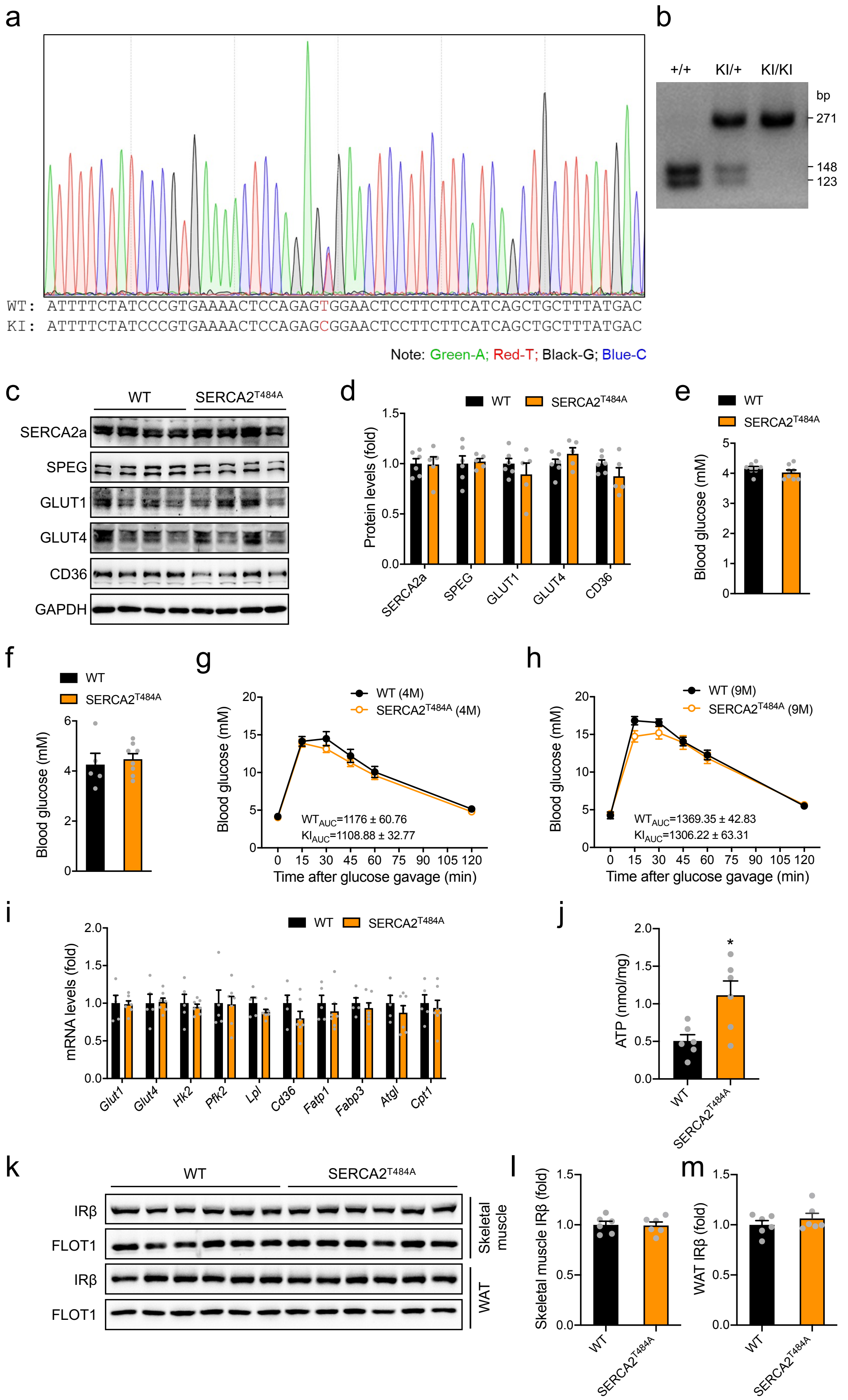

Supplementary Figure S3

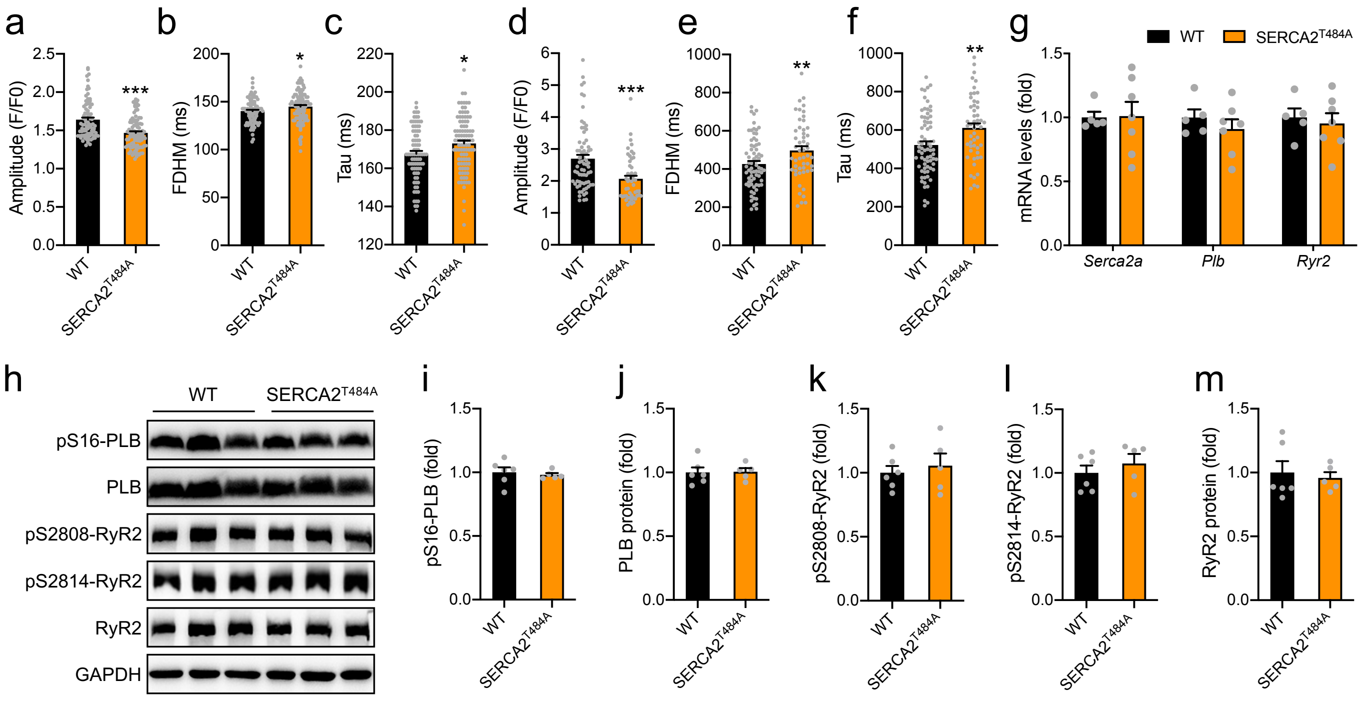

Supplementary Figure S4

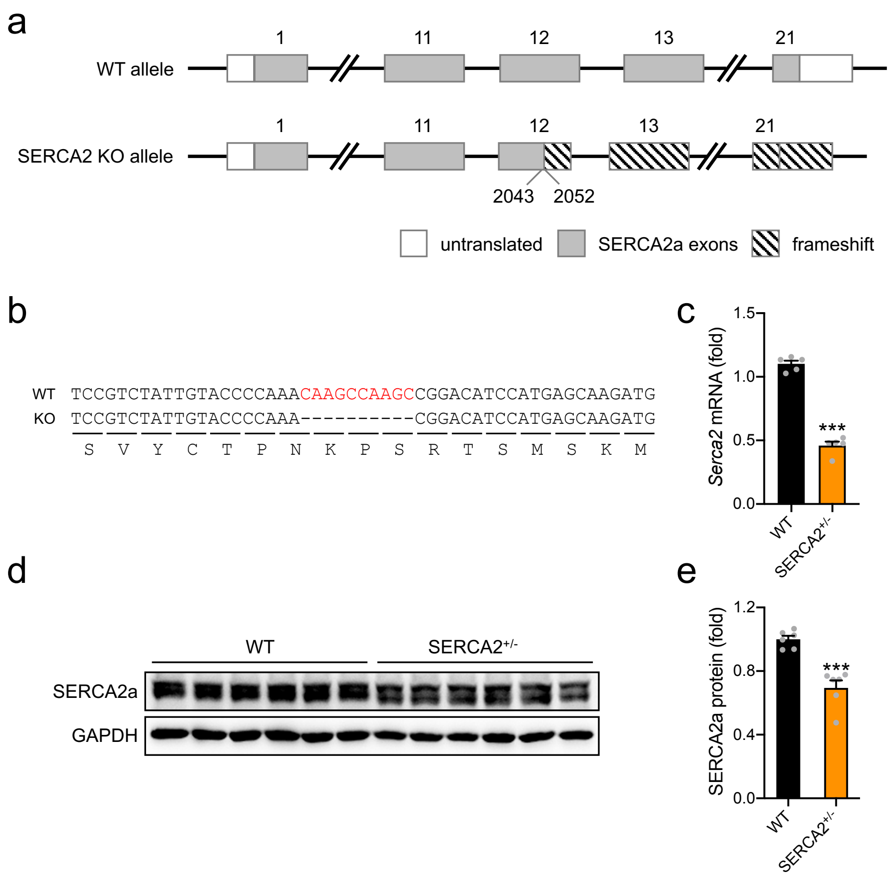

Supplementary Figure S5

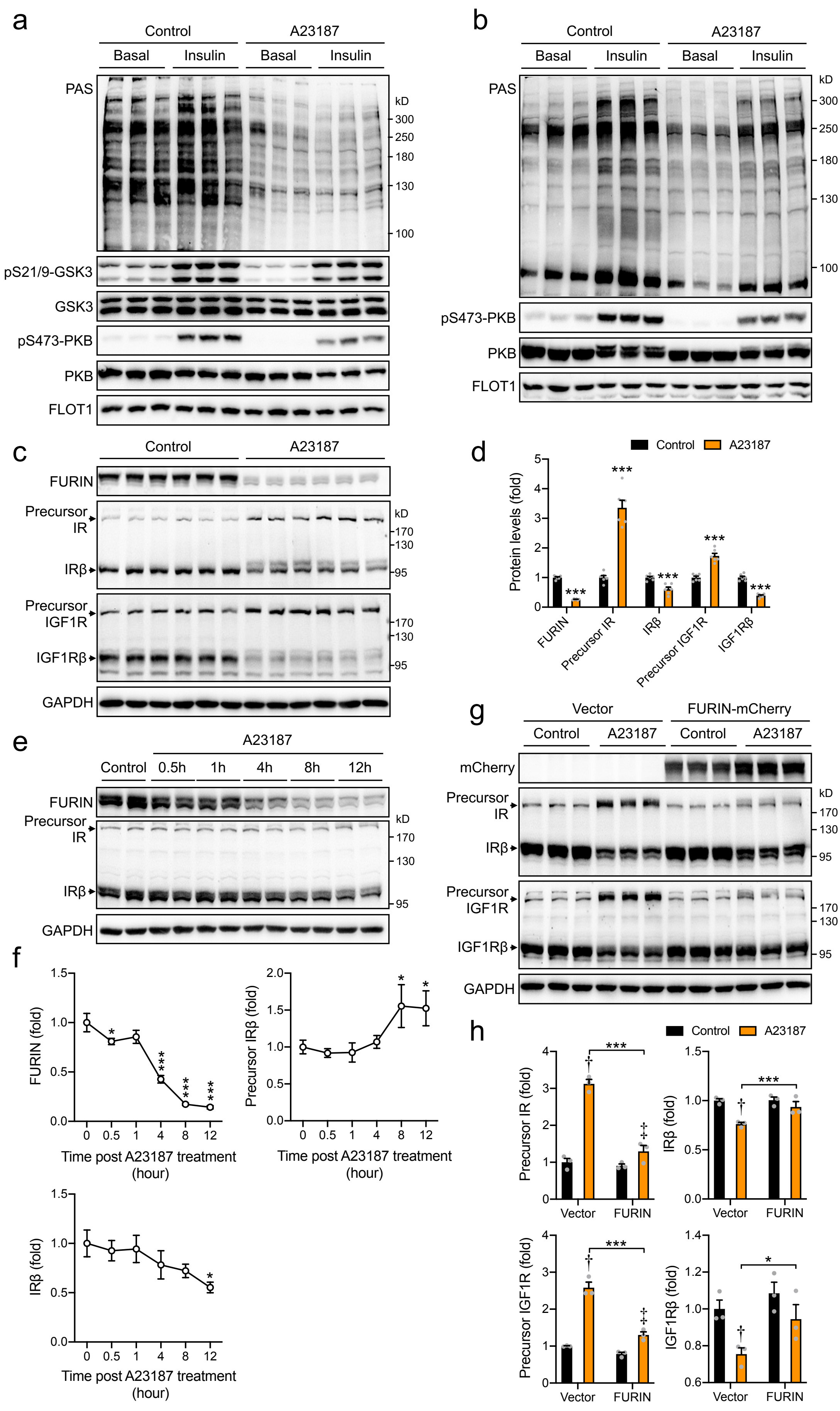

Supplementary Figure S6

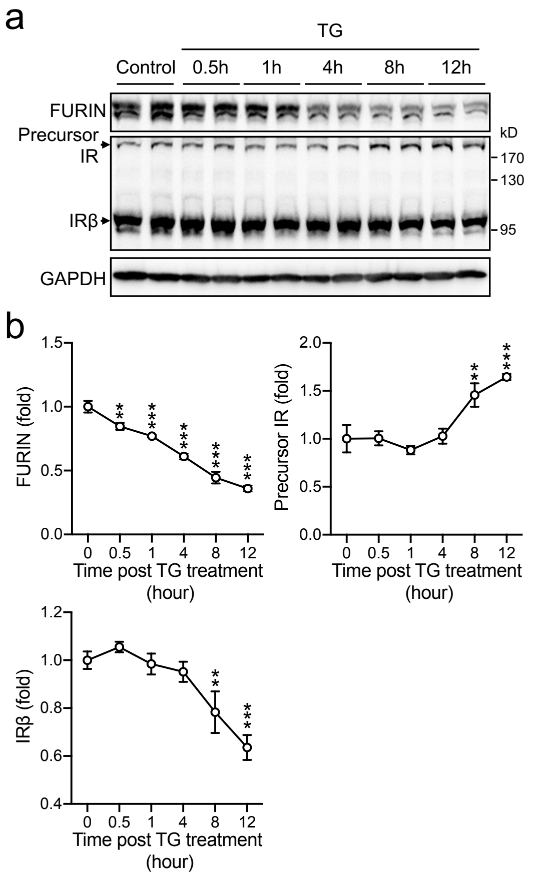

Supplementary Figure S7

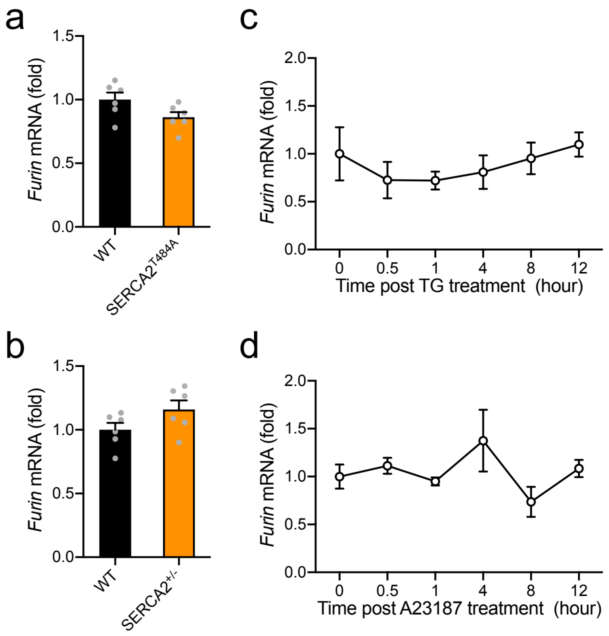

Supplementary Figure S8

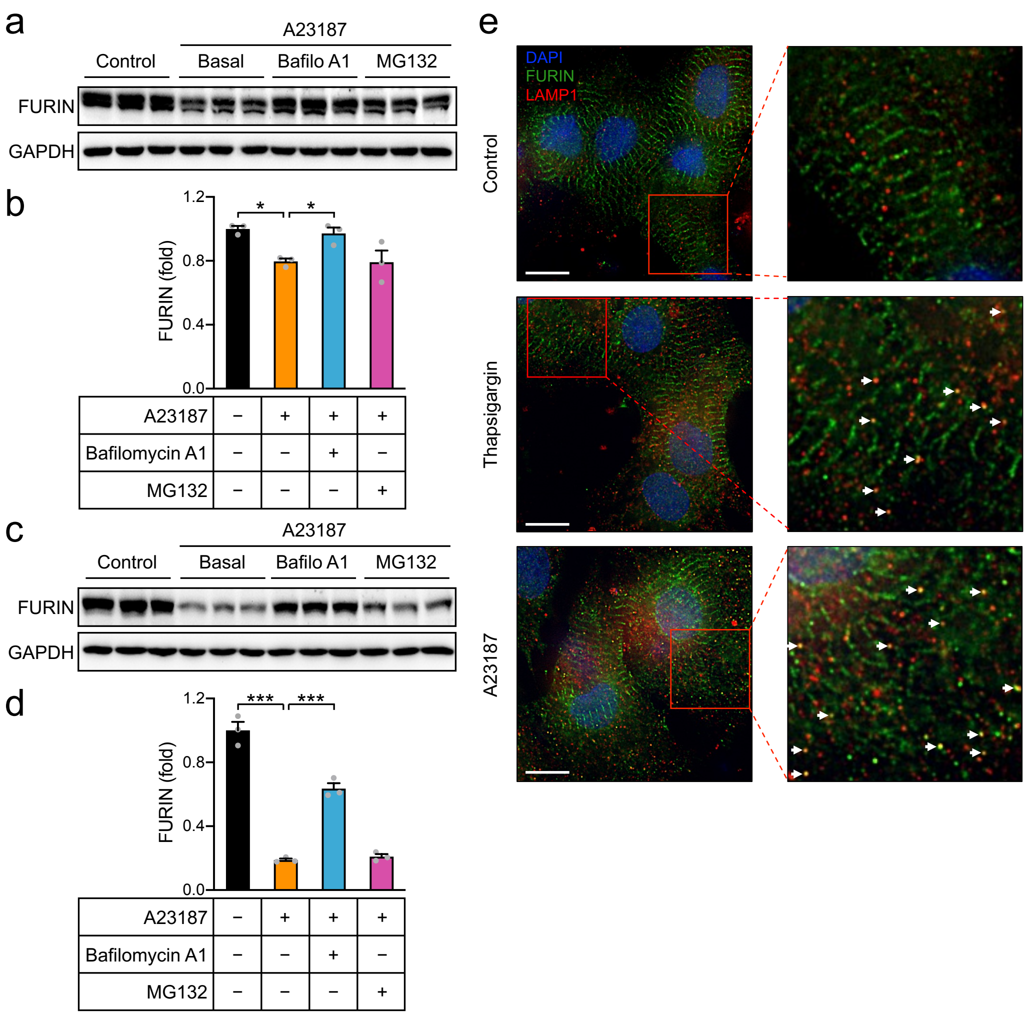

Supplementary Figure S9

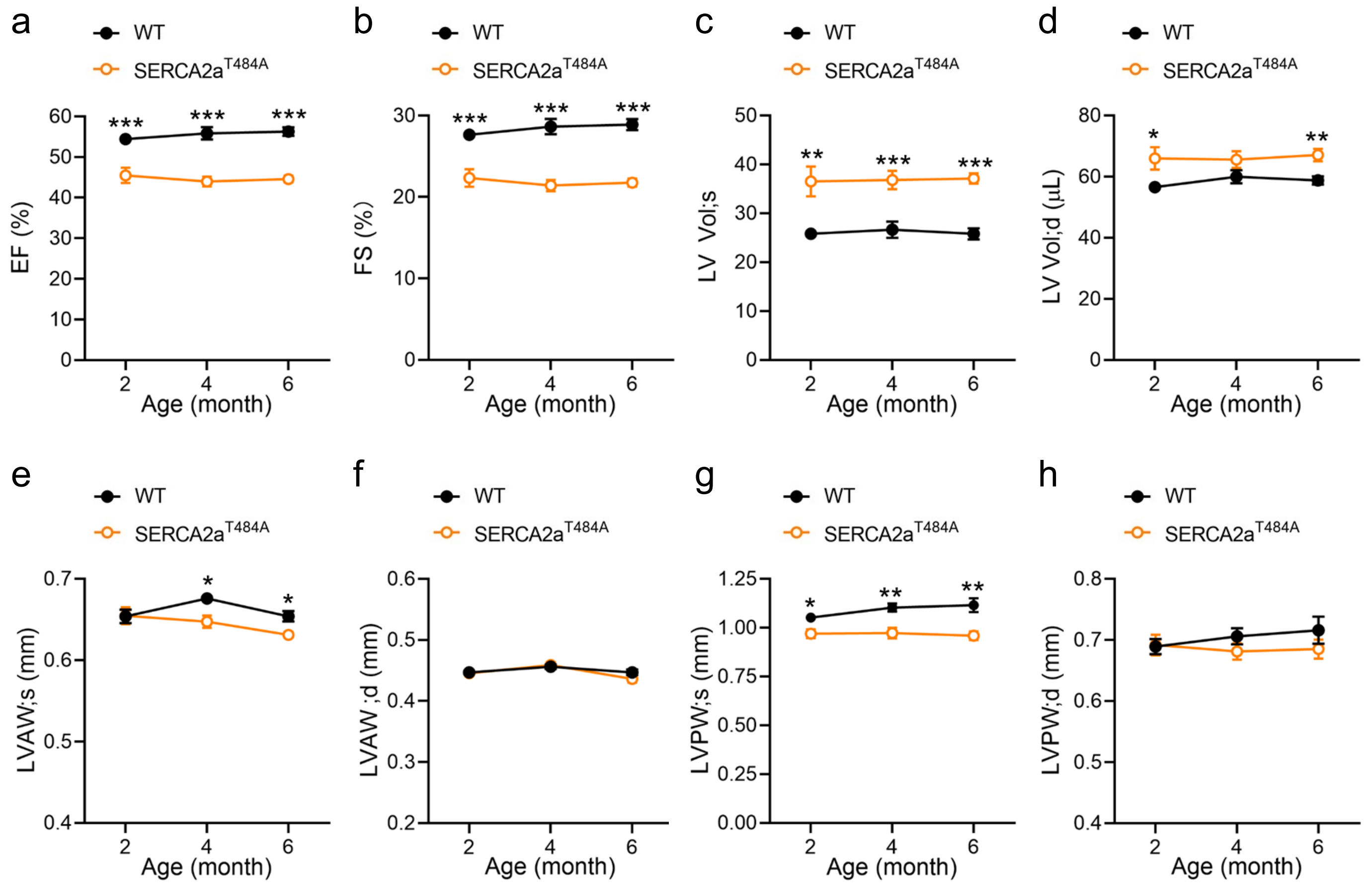

Supplementary Figure S10

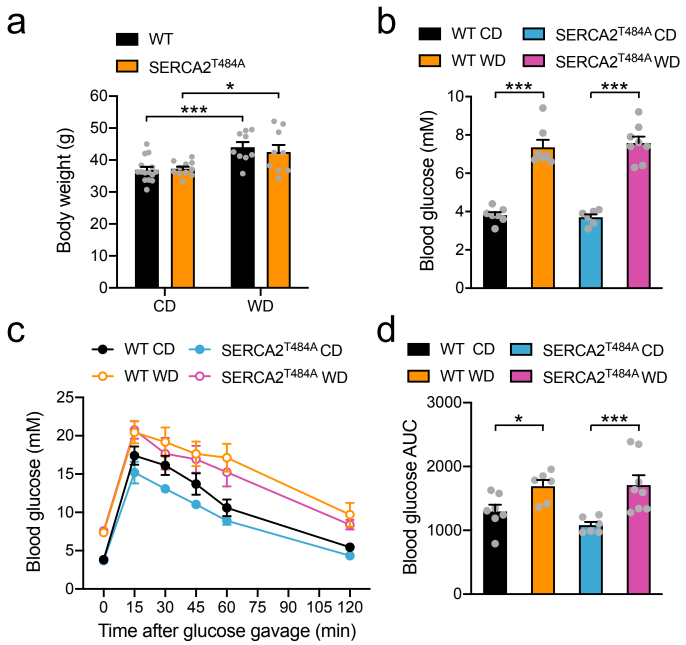

Supplement: loac013_suppl_Supplementary_Figures [file loac013_suppl_Supplementary_Figures.pdf]
